# Artificial intelligence for detection of microsatellite instability in colorectal cancer—a multicentric analysis of a pre-screening tool for clinical application

**DOI:** 10.1016/j.esmoop.2022.100400

**Published:** 2022-03-02

**Authors:** A. Echle, N. Ghaffari Laleh, P. Quirke, H.I. Grabsch, H.S. Muti, O.L. Saldanha, S.F. Brockmoeller, P.A. van den Brandt, G.G.A. Hutchins, S.D. Richman, K. Horisberger, C. Galata, M.P. Ebert, M. Eckardt, M. Boutros, D. Horst, C. Reissfelder, E. Alwers, T.J. Brinker, R. Langer, J.C.A. Jenniskens, K. Offermans, W. Mueller, R. Gray, S.B. Gruber, J.K. Greenson, G. Rennert, J.D. Bonner, D. Schmolze, J. Chang-Claude, H. Brenner, C. Trautwein, P. Boor, D. Jaeger, N.T. Gaisa, M. Hoffmeister, N.P. West, J.N. Kather

**Affiliations:** 1 Department of Medicine III, University Hospital RWTH Aachen, Aachen, Germany; 2 Division of Pathology and Data Analytics, Leeds Institute of Medical Research at St James’s, University of Leeds, Leeds, UK; 3 Department of Pathology, GROW School for Oncology and Developmental Biology, Maastricht University Medical Center+, Maastricht, The Netherlands; 4 Department of Epidemiology, Maastricht University Medical Center+, Maastricht, The Netherlands; 5 Department of Abdominal and Transplantation Surgery, University Hospital of Zurich, Zurich, Switzerland; 6 Department of Surgery, Universitätsmedizin Mannheim, Medical Faculty Mannheim, Heidelberg University, Mannheim, Germany; 7 Division of Thoracic Surgery, Academic Thoracic Center Mainz, University Medical Center Mainz, Johannes Gutenberg University Mainz, Mainz, Germany; 8 Department of Medicine II, University Medical Center Mannheim, Medical Faculty Mannheim, Heidelberg University, Mannheim, Germany; 9 Mannheim Institute for Innate Immunoscience (MI3) and Clinical Cooperation Unit Healthy Metabolism, Center of Preventive Medicine and Digital Health, Medical Faculty Mannheim, Heidelberg University, Mannheim, Germany; 10 Mannheim Cancer Center, Medical Faculty Mannheim, Heidelberg University, Mannheim, Germany; 11 Division of Signaling and Functional Genomics, German Cancer Research Center (DKFZ), Heidelberg, Germany; 12 Institut für Pathologie Charité, Berlin, Germany; 13 Division of Clinical Epidemiology and Aging Research, German Cancer Research Center, Heidelberg, Germany; 14 Digital Biomarkers for Oncology Group, National Center for Tumor Diseases (NCT), German Cancer Research Center (DKFZ), Heidelberg, Germany; 15 Institute of Pathology, Inselspital, University of Bern, Bern, Switzerland; 16 Gemeinschaftspraxis Pathologie, Starnberg, Germany; 17 Clinical Trial Service Unit, University of Oxford, Oxford, UK; 18 Center for Precision Medicine and Department of Medical Oncology, City of Hope National Medical Center, Duarte, USA; 19 Department of Pathology, City of Hope Comprehensive Cancer Center, Duarte, USA; 20 Department of Community Medicine & Epidemiology, Lady Davis Carmel Medical Center, Ruth & Bruce Rappaport Faculty of Medicine, Technion-Israel Institute of Technology, Haifa, Israel; 21 Steve and Cindy Rasmussen Institute for Genomic Medicine, Lady Davis Carmel Medical Center and Technion Faculty of Medicine, Clalit National Cancer Control Center, Haifa, Israel; 22 Division of Cancer Epidemiology, German Cancer Research Center (DKFZ), Heidelberg, Germany; 23 Cancer Epidemiology Group, University Cancer Center Hamburg, University Medical Center Hamburg-Eppendorf, Hamburg, Germany; 24 Division of Preventive Oncology, German Cancer Research Center (DKFZ) and National Center for Tumor Diseases (NCT), Heidelberg, Germany; 25 German Cancer Consortium (DKTK), German Cancer Research Center (DKFZ), Heidelberg, Germany; 26 Institute of Pathology, University Hospital RWTH Aachen, Aachen, Germany; 27 Department of Nephrology and Immunology, University Hospital RWTH Aachen, Aachen, Germany; 28 Medical Oncology, National Center for Tumor Diseases (NCT), University Hospital Heidelberg, Heidelberg, Germany

**Keywords:** artificial intelligence, biomarker, colorectal cancer, microsatellite instability, Lynch syndrome, deep learning

## Abstract

**Background:**

Microsatellite instability (MSI)/mismatch repair deficiency (dMMR) is a key genetic feature which should be tested in every patient with colorectal cancer (CRC) according to medical guidelines. Artificial intelligence (AI) methods can detect MSI/dMMR directly in routine pathology slides, but the test performance has not been systematically investigated with predefined test thresholds.

**Method:**

We trained and validated AI-based MSI/dMMR detectors and evaluated predefined performance metrics using nine patient cohorts of 8343 patients across different countries and ethnicities.

**Results:**

Classifiers achieved clinical-grade performance, yielding an area under the receiver operating curve (AUROC) of up to 0.96 without using any manual annotations. Subsequently, we show that the AI system can be applied as a rule-out test: by using cohort-specific thresholds, on average 52.73% of tumors in each surgical cohort [total number of MSI/dMMR = 1020, microsatellite stable (MSS)/ proficient mismatch repair (pMMR) = 7323 patients] could be identified as MSS/pMMR with a fixed sensitivity at 95%. In an additional cohort of *N* = 1530 (MSI/dMMR = 211, MSS/pMMR = 1319) endoscopy biopsy samples, the system achieved an AUROC of 0.89, and the cohort-specific threshold ruled out 44.12% of tumors with a fixed sensitivity at 95%. As a more robust alternative to cohort-specific thresholds, we showed that with a fixed threshold of 0.25 for all the cohorts, we can rule-out 25.51% in surgical specimens and 6.10% in biopsies.

**Interpretation:**

When applied in a clinical setting, this means that the AI system can rule out MSI/dMMR in a quarter (with global thresholds) or half of all CRC patients (with local fine-tuning), thereby reducing cost and turnaround time for molecular profiling.

## Highlights

• We describe the largest multicentric study of AI in molecular cancer diagnostics to date.
• Our open-source AI system achieves a high pre-screening performance for detection of MSI.
• Our data highlight the role of large-scale validation of AI biomarkers in different geographic and ethnic populations.

## Introduction

Microsatellite instability (MSI) is a biomarker of high clinical importance in colorectal cancer (CRC).^1^ MSI is measured with polymerase chain reaction (PCR) or next-generation sequencing in tissue samples. In clinical practice, MSI is virtually synonymous with mismatch repair deficiency (dMMR), which is measured by immunohistochemistry (IHC) and shows a high concordance with MSI.^2^ MSI/dMMR [the opposite of which is microsatellite stability (MSS) or proficient mismatch repair (pMMR)] changes treatment in all stages of CRC. In locally resectable cases, MSI/dMMR status is one of the multiple factors which decides whether patients should receive adjuvant chemotherapy after surgery.^2^ In metastatic disease, the presence of MSI/dMMR makes patients eligible for immunotherapy with checkpoint inhibitors.^3^ Finally, testing for MSI/dMMR is used as the first test in a sequence of screening tests for Lynch syndrome, one of the most common hereditary tumor syndromes.^4^ MSI/dMMR should be tested in every CRC patient according to the UK National Institute for Health and Care Excellence (NICE) guidelines.^5^ Approximately 12% of all CRC patients have sporadic MSI/dMMR and 2%-4% of all CRC patients have MSI/dMMR due to Lynch syndrome.^6^^,^^7^ Since 2019, >10 studies have shown that MSI/dMMR status can be detected from digitized pathology slides stained with hematoxylin and eosin (H&E).^8^, ^9^, ^10^, ^11^, ^12^, ^13^, ^14^, ^15^, ^16^, ^17^ The key technology that enables this is deep learning (DL), an artificial intelligence (AI) method. Such AI-based systems for detection of MSI/dMMR status from routine histopathology slides are also in the focus of commercial interest, as evident by a US patent application of this technology (#16/412362 filed on 2019-11-14 by Tempus Labs). As routine pathology diagnostic workflows are expected to become fully digital, AI-based biomarkers could be inexpensively implemented in existing workflows.^18^ In this context, MSI/dMMR detection with AI could be a blueprint for other biomarkers.^19^ Clinical use of these AI biomarkers requires extensive validation steps.^20^ In addition, it is still unclear how AI-based MSI/dMMR testing performs across different populations of patients. Furthermore, many previous studies have reported continuous prediction scores (an MSI/dMMR probability) for individual patients.^8^, ^9^, ^10^ Yet, for clinical use, the AI system should also be able to make the call whether the patient should undergo gold-standard testing for confirmation in addition to giving a probability score. In particular, previous studies have suggested that AI-based MSI/dMMR testing could be used as a pre-screening tool to select candidates for gold-standard testing and rule out the remaining patients.^9^^,^^10^ However, it is unclear how many patients exactly could be ruled out in clinical routine by such a system when applied to large and diverse populations of CRC patients. In addition, it is unclear if clinical-grade performance can be achieved on biopsies.^10^ In the present study, we systematically investigated these aspects by using a large collection of nine patient cohorts of CRC surgical specimens and one cohort of CRC endoscopic biopsies.

## Materials and methods

### Ethics statement

This study was carried out in accordance with the Declaration of Helsinki. This study is a retrospective analysis of digital images of anonymized archival tissue samples of multiple cohorts of CRC patients. Data were collected and anonymized, and ethics approval was obtained at each contributing center (Supplementary Table S1, available at https://doi.org/10.1016/j.esmoop.2022.100400).

### STARD checklist

In this study we covered all the items in the STAndards for Reporting Diagnostic accuracy studies (STARD) for the transparency of the reported results. Supplementary Table S2, available at https://doi.org/10.1016/j.esmoop.2022.100400 shows these items and their corresponding covering page in this current study.

### Cohort description

Through coordination by the MSIDETECT consortium (www.msidetect.eu), we collected H&E tissue slides from *N* = 8343 colorectal cancer patients from nine patient cohorts (Supplementary Table S3, available at https://doi.org/10.1016/j.esmoop.2022.100400) as follows: Darmkrebs: Chancen der Verhütung durch Screening, Southwest Germany (DACHS)^21^^,^^22^ is a large population-based case-control and patient cohort study on CRC, including samples of patients with stages I-IV from different laboratories in southwestern Germany coordinated by the German Cancer Research Center (Heidelberg, Germany). QUASAR is the ‘Quick and Simple and Reliable’ trial (Yorkshire, UK), which investigated survival under adjuvant chemotherapy in patients from the UK with mostly stage II tumors.^23^^,^^24^ The public repository ‘The Cancer Genome Atlas’, (TCGA, publicly available at https://portal.gdc.cancer.gov/, USA)^25^^,^^26^ included tumors of all stages with the primary intent of genomic characterization. The Netherlands Cohort Study, The Netherlands (NLCS)^27^^,^^28^ comprised tissue samples as part of the Rainbow-TMA consortium, and like DACHS, this study included patients with any tumor stage. Yorkshire Cancer Research Bowel Cancer Improvement Programme, (YCR-BCIP), Yorkshire, UK is a population-based register of bowel cancer patients in the Yorkshire region of the UK.^4^^,^^29^ The DUSSEL (Düsseldorf, Germany) cohort is a case series of CRC resected in curative intent and collected at the Marien-Hospital in Duesseldorf, Germany, between January 1990 and December 1995 with the intent of performing translational research studies.^30^ The Molecular Epidemiology of Colorectal Cancer, (MECC) study, Israel^31^ is a population-based case-control study in northern Israel. University Medical Center Mannheim, (UMM), Germany, is a multicentric collection of CRC in patients with inflammatory bowel diseases, centrally collected in Mannheim, Germany, with contributions from medical centers in Germany. The MUNICH (Munich, Germany) CRC series is a case series collected with translational research intent at the Technical University of Munich in Germany. Detailed clinicopathological variables are shown in Supplementary Table S3, available at https://doi.org/10.1016/j.esmoop.2022.100400. For each patient, either an MSI or a dMMR status, obtained with PCR or IHC, respectively, was available (Supplementary Table S3, available at https://doi.org/10.1016/j.esmoop.2022.100400). Although MSI status and dMMR status are not 100% concordant,^4^ they are interchangeably used in clinical settings and in this study. From all cohorts, formalin-fixed paraffin-embedded tissue was used—only in the TCGA cohort, a small number of frozen sections were present in the slide set labeled as ‘diagnostic slides’. Slides were scanned decentrally at the respective centers. Supplementary Figures S1-10, available at https://doi.org/10.1016/j.esmoop.2022.100400, show the consort charts for all the cohorts selected for this study. The patients are dropped out from further analysis in case of missing MSI∖dMMR status, missing of whole slide image (WSI) or because of the preprocessing step.

### Experimental setup

To estimate the performance of AI-based detection of MSI/dMMR in each of the nine cohorts, we employed a novel experimental design: Leave-one-cohort-out cross-validation. This means that we trained a neural network for detection of MSI/dMMR status on all patients from eight out of nine cohorts and tested it on all patients in the ninth cohort. Each cohort was used as a test cohort exactly once. Thereby, we trained nine distinct deep neural networks in this study (for external validation) and additional networks for within-cohort cross-validation. Ultimately, this design ensured that MSI/dMMR detections were available for every single patient. No patient was ever part of the training process and validation process at any point. In this way the performance of DL-based MSI/dMMR detection in nine different external validation sets can be analyzed and compared. This leave-one-cohort-out cross-validation was compared to a threefold patient-level within-cohort cross-validation approach which was run separately on each cohort.

### Image preprocessing and DL

All data used in this study were preprocessed according to the ‘Aachen protocol for Deep Learning histopathology’.^32^ All digitized WSIs were tessellated into image tiles of 256 μm edge length saved at 512 pixel edge length. No manual annotations of tumor tissue were used. Tiles were generated from the full non-annotated WSI. Image tiles containing background or blurry ones were automatically removed from the dataset during this process using the detected edge quantity (canny edge detection in Python’s OpenCV package). Specifically, we obtained a normalized edge image (using cv.canny; OpenCV, https://opencv.org/) and then removed all tiles with a mean value below a threshold of 4. All image tiles were subsequently color-normalized to a target image (https://github.com/jnkather/DeepHistology/blob/master/subroutines_normalization/Ref.png) with the Macenko method.^33^ Subsequently, a ResNet18 neural network model which was pre-trained on the Imagenet database was re-trained on all image tiles from all patients in the training set. The high performance of ResNet18 in computational pathology tasks has been shown in previous work.^8^ For all patients who had >500 tiles, only 500 randomly selected tiles were used for all subsequent steps. Furthermore, to balance the number of tiles in the MSI/dMMR and MSS/pMMR class in the training set, the tiles in the more abundant group were randomly undersampled just before training to achieve class balance. No such undersampling step was carried out for the tiles in the test set. For processing by the neural network (training and deployment), all tiles were resized to 224 × 224 pixels. The weights in the last half layers of the pre-trained network were updated for five epochs (mini-batch size of 1024), with a fixed learning rate of 1e-4 (weight decay of 1e-5), while the weights of the first half layers were frozen during the training. Adam optimizer and cross-entropy loss function were used to fine-tune the weights of the model. Patient-level scores were obtained by calculating the fraction of tiles predicted to be of each class by using a tile-level threshold of 0.5, relative to the total number of tiles per patient. The overall workflow and the hyperparameters were previously validated in other studies in colorectal and gastric cancer.^8^^,^^10^^,^^34^

### Statistical analyses

We pursued multiple approaches to determine the optimal threshold on the test set: First, we obtained a ‘cohort-specific threshold’ at fixed 95% sensitivity in each test set. Second, we tested three candidates for a global threshold: 0.25, 0.50 and 0.75 which were subsequently applied to all cohorts. Third, we trained the DL system on all eight training cohorts, obtained a ‘learned threshold’ by averaging the optimal thresholds of all eight training cohorts (which were obtained by cross-validation within each cohort, and finally deployed the model and the learned threshold to the test cohort (the ninth cohort). Statistical endpoints were the area under the receiver operating curve (AUROC), sensitivity, specificity, positive predictive value, negative predictive value and F1-score. We calculated the 95% confidence intervals of the area-under-the-curve values which are formed from quantiles of the 1000-bootstrap resampling distribution.

### Reader study

To identify potential reasons for misclassified cases, we carried out a reader study of misclassified [false-positive (FP) and false-negative (FN)] samples. Two pathologists (SFB, NPW) reviewed FN and FP cases at different thresholds but were blinded to other clinicopathological features. For each case, they commented on the presence of potential technical confounders (artifacts), amount of tumor tissue on the slide and unusual or rare morphological patterns. Among these potential confounders, the pathologists chose the most relevant confounder, which was then used for further statistical analysis.

### Implementation, code availability and data availability

All codes were implemented in Python 3.8 (Python Software Foundation, Wilmington, DE) using Pytorch and were run on Windows Server 2019 on multiple NVIDIA RTX8000 graphics-processing units. All source codes for preprocessing are available at https://github.com/KatherLab/preProcessing and all source codes for DL are available as part of the ‘Histology Image Analysis’ (HIA) package at https://github.com/KatherLab/HIA. All trained classifiers are available at https://doi.org/10.5281/zenodo.5151502 and are available for re-use. Access to raw data can be requested from the respective consortia who independently decide on data access for their respective patient cohorts. The corresponding authors of this study do not have any role in decisions about data access in the primary datasets.

## Results

### DL achieves robust MSI/dMMR detection performance across cohorts

**Figure 1:**
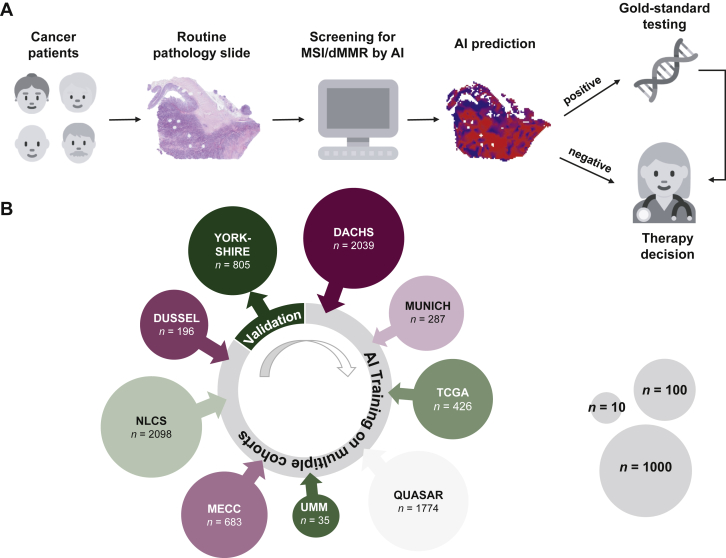
**Study overview.** (A) Detection of microsatellite instability by deep learning could be used as a screening tool and rule out a part of the patients from further testing. (B) A deep learning classifier was trained on eight out of nine cohorts and validated in the remaining one. Circle size indicates the patient number of each cohort as shown. Icon source: Twitter Twemoji (CC-BY 4.0). AI, artificial intelligence; dMMR, mismatch repair deficiency; DACHS, Darmkrebs: Chancen der Verhütung durch Screening; MECC, Molecular Epidemiology of Colorectal Cancer; MSI, microsatellite instability; NLCS, Netherlands Cohort Study; QUASAR, Quick and Simple and Reliable; TCGA, The Cancer Genome Atlas; UMM, University Medical Center Mannheim.

In this study, a DL system was used to detect MSI/dMMR status from routine digitized H&E tissue slides of CRC (Figure 1A) in a novel leave-one-cohort-out experimental design (Figure 1B). We found that MSI/dMMR status could be determined from histology slides with a high performance: eight out of nine cohorts achieved a patient-level AUROC of >0.85 (Table 1), corresponding to a high degree of separation of the detected MSI/dMMR scores in the ‘true MSI/dMMR’ compared to the ‘true MSS/pMMR’ group (Supplementary Figure S11, available at https://doi.org/10.1016/j.esmoop.2022.100400). In the YCR-BCIP cohort, the MSI/dMMR detection AUROC was 0.96 (0.94-0.98). Only one cohort achieved lower results: in the MECC cohort, the AUROC was 0.74 (0.69-0.80). The performance on held-out validation cohorts was comparable to the within-cohort performances (Table 1), demonstrating the robustness of classifiers trained in a multicentric setting. Supplementary Figures S12 and S13, available at https://doi.org/10.1016/j.esmoop.2022.100400 show the ROC curves and precision-recall curves correspondingly for all the internal and external validation experiments.

**Table 1:** Results of external validation of the MSI detection network in all cohorts

Cohort	*N* patients included	AUROC deployment (95% CI)	Specificity at 95% sensitivity (%)	Positive predictive value (%)	Negative predictive value (%)	Rule-out fraction	False-negative fraction	F1-score	AUROC within-cohort (95% CI)
DACHS	2039	0.89 (0.87-0.92)	50	18	99	0.459	0.005	0.30	0.91 (0.88-0.93)
QUASAR	1774	0.93 (0.91-0.95)	71	34	99	0.614	0.008	0.50	0.90 (0.88-0.92)
TCGA	426	0.91 (0.87-0.95)	53	25	98	0.465	0.007	0.40	0.79 (0.72-0.85)
NLCS	2098	0.92 (0.90-0.94)	69	27	99	0.622	0.005	0.42	0.85 (0.82-0.87)
YCR-BCIP	805	0.96 (0.94-0.98)	89	58	99	0.768	0.007	0.72	0.93 (0.90-0.96)
DUSSEL	196	0.85 (0.74-0.93)	28	15	96	0.230	0.010	0.26	0.75 (0.64-0.85)
MECC	683	0.74 (0.69-0.80)	15	17	93	0.126	0.009	0.29	0.70 (0.64-0.75)
UMM	35	0.92 (0.69-1.00)	68	23	95	0.629	0.029	0.35	0.98 (0.93-1.00)
MUNICH	287	0.88 (0.80-0.95)	40	17	98	0.352	0.007	0.29	0.80 (0.71-0.88)

Statistics describe the cohort-specific threshold when the network was trained in all cohorts except the one it was tested on. As a comparison, the last column compared this validation performance to the results for threefold cross-validation experiments within each cohort. A classifier used for pre-screening should have a high true-negative fraction (rule-out fraction) and a low false-negative fraction. For detailed patient numbers, see CONSORT flowcharts in Supplementary Figures S1-9, available at https://doi.org/10.1016/j.esmoop.2022.100400.

AUROC, area under the receiver operating curve; CI, confidence interval; DACHS, Darmkrebs: Chancen der Verhütung durch Screening; MECC, Molecular Epidemiology of Colorectal Cancer; NLCS, Netherlands Cohort Study; QUASAR, Quick and Simple and Reliable; TCGA, The Cancer Genome Atlas; UMM, University Medical Center Mannheim; YCR-BCIP, Yorkshire Cancer Research Bowel Cancer Improvement Programme.

### DL can rule out patients based on cohort-specific thresholds

**Figure 2:**
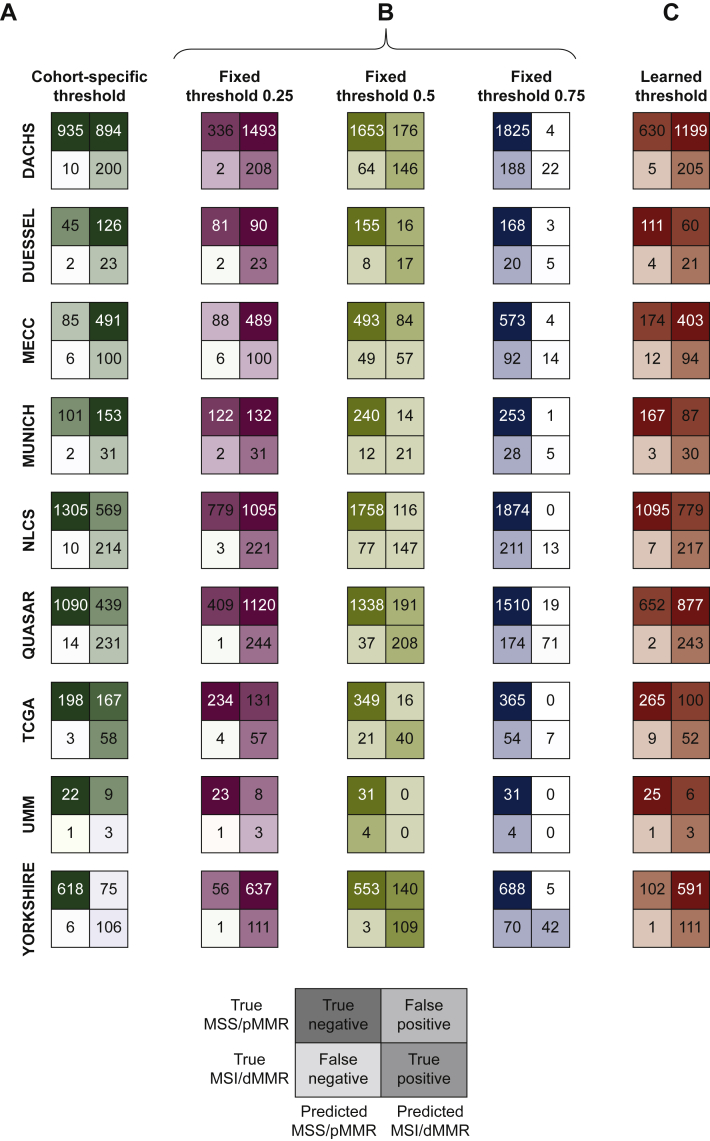
**Test statistics for the pre-screening tool.** Test performance in the different cohorts displayed as patients classified true/false positive/negative by the deep learning classifier based on cohort-specific thresholds (calculated individually for each test dataset), fixed thresholds (0.25, 0.5 and 0.75) and learned thresholds (averaged thresholds of the eight training datasets used for the corresponding experiment). Cohort-specific and learned thresholds are listed in Supplementary Table S4, available at https://doi.org/10.1016/j.esmoop.2022.100400. dMMR, mismatch repair deficiency; DACHS, Darmkrebs: Chancen der Verhütung durch Screening; MECC, Molecular Epidemiology of Colorectal Cancer; MSI, microsatellite instability; MSS, microsatellite stable; NLCS, Netherlands Cohort Study; TCGA, The Cancer Genome Atlas; pMMR, proficient mismatch repair; QUASAR, Quick and Simple and Reliable; UMM, University Medical Center Mannheim.

While an AUROC gives an estimate of classifier performance, real-world use of classifiers requires a ‘threshold’ that converts probabilities into binary predictions. Therefore, for each dataset, we derived a cohort-specific threshold value from the model predictions. This threshold was set at a 95% sensitivity value, aiming to minimize the fraction of FN predictions. We found that for such a (95%) threshold, negative predictive values were >93% in all cohorts (Table 1), demonstrating the potential of this AI system as a pre-screening tool. The true-negative fraction or rule-out fraction (how many patients can be safely excluded from confirmatory testing) ranged from 12.6% in MECC to 78.8% in YCR-BCIP while the ‘FN fraction’ remained always <0.3% (Table 1, Figure 2).

### Performance of predefined threshold values for patient classification

**Figure 3:**
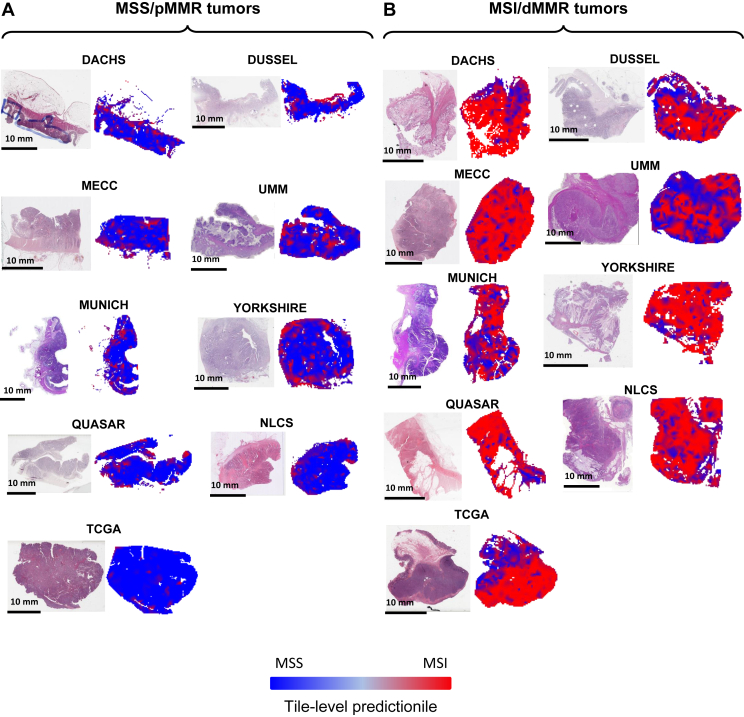
**Representative hematoxylin–eosin (H&E) tissue slides**. Patients with (A) MSS/pMMR and (B) MSI/dMMR tumors from all cohorts and corresponding detection maps based on the tile-wise detection of the deep learning classifier. dMMR, mismatch repair deficiency; DACHS, Darmkrebs: Chancen der Verhütung durch Screening; MECC, Molecular Epidemiology of Colorectal Cancer; MSI, microsatellite instability; MSS, microsatellite stable; NLCS, Netherlands Cohort Study; TCGA, The Cancer Genome Atlas; pMMR, proficient mismatch repair; QUASAR, Quick and Simple and Reliable; UMM, University Medical Center Mannheim.

An alternative to cohort-specific determination of optimal thresholds is the use of fixed thresholds for MSI/dMMR classification in patients. We defined 0.25, 0.50 and 0.75 as cutoff values for determining which patients to rule out. Using the lowest threshold (0.25), *n* = 2128 (25.5%) patients were correctly classified as MSS/pMMR while only 22 patients (0.26%) were incorrectly detected to be MSS/pMMR (false negatives). Thus, this high-sensitivity classifier which is not tuned to specific cohorts would be able to exclude 25.5% of all patients from subsequent molecular testing, while only missing 0.26% of all patients if it is used as a pre-screening test before ultimate gold-standard testing (Figure 2B), using a fixed threshold value which does not need to be tailored to a specific cohort. To analyze reasons for the high classification performance, we reviewed spatial detection maps generated by the DL classifiers (Figure 3). This qualitative analysis was carried out in at least 10 tumors per cohort by two observers (NGL, JNK). We found that in MSS/pMMR tumors (as defined by the ground truth method), the classifier was—in the majority of cases—homogeneously detecting tumor tissue and adjacent non-tumor tissue to be MSS/pMMR. Interestingly, the tumor-invasive edge was detected to be MSI/dMMR in approximately half of all analyzed cases, presumably due to tumor-adjacent lymphocytes (Figure 3A). In true MSI/dMMR tumors (as defined by the ground truth), the tumor tissue was generally homogeneously detected to be MSI/dMMR (Figure 3B). Subsequently, we carried out a systematic analysis of misclassified cases.

### Learned thresholds yield a high performance

Cohort-specific thresholds indicate a potential for high performance of the classifier, but can lead to overfitting while fixed thresholds can lead to underperformance. Therefore, we investigated an additional ‘learned threshold’ which was determined in the training cohorts and applied to each testing cohort (Supplementary Table S4, available at https://doi.org/10.1016/j.esmoop.2022.100400). We found that this threshold yielded a high performance across all cohorts as evident by a low number of FN cases and a high number of true-negative cases, representing a high rule-out-fraction (Figure 2C). As we can see in Supplementary Table S4, available at https://doi.org/10.1016/j.esmoop.2022.100400, the average of these learned thresholds for all the nine cohorts is 0.29 which can be defined as an optimal threshold for all the cohorts.

### Clinical, molecular and morphological properties of misclassified cases

Next, we investigated the clinical and molecular properties of misclassified cases in the TCGA cohort, specifically, patients who were assigned high MSI/dMMR scores but were MSS/pMMR according to gold-standard methods. Sixteen out of 365 MSS/pMMR patients were assigned MSI/dMMR probability scores >0.5, while in the true MSI/dMMR patients, 41 out of 61 patients achieved scores of >0.5. Among these 16 patients, 11 patients had a known consensus molecular subtype (CMS) and 6 out of 11 patients were CMS1, which is highly associated with an MSI-like inflamed tumor microenvironment.^35^ In contrast, only 52 out of 368 patients in the overall cohort were CMS1. Furthermore, 2 out of 16 patients were hypermutated according to a predefined criterion^36^ while 73 out of 460 patients in the overall cohort were hypermutated. Lastly, 15 out of 16 patients had detailed primary tumor location data available and 10 out of these 15 patients were right-sided primary tumors while 176 out of 424 patients in the overall cohort had right-sided primary tumors (Supplementary Figure S14, available at https://doi.org/10.1016/j.esmoop.2022.100400).

**Figure 4:**
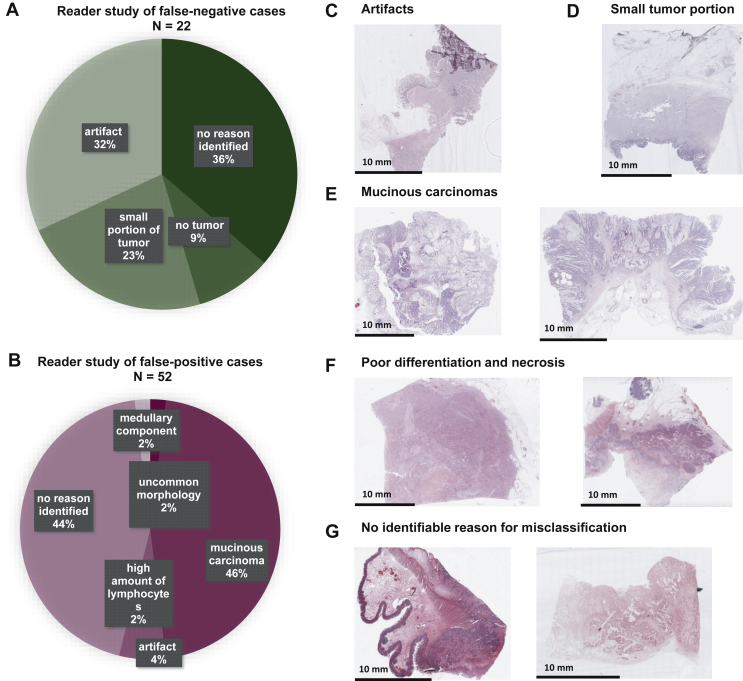
**Result of the reader study of misclassified cases.** (A and B) Possible reasons for misclassification identified by a pathologist and their incidence in false-positive and false-negative cases. (C-G) Example tissue slides for different categories in the reader study.

Subsequently, we extended the analysis of misclassified cases to FPs and FNs in all cohorts. Specifically, we carried out a histopathological review of all FN cases based on a fixed threshold of 0.25 and of all FP cases based on a fixed threshold of 0.75 (Figure 2B). An exception needed to be made for the TCGA cohort, as there were no FP cases at a threshold of 0.75 and therefore, FP cases at a threshold of 0.5 were reviewed. Readers concluded that in the FP group, a plausible reason for misclassifications could be identified in 56% of cases (Figure 4A), mostly related to mucinous differentiation of the tumor, which in the observed cases was associated with a low percentage of tumor epithelium on the tissue slides. In 23 out of 52 (44%) misclassified FP cases, no reason was identifiable. In a clinical application as a pre-screening tool, FN cases are more problematic than FP cases. In the FN cohort, a probable reason was identified in 15 out of 22 (68%) cases. The most common reasons were artifacts on the slides (32%, especially poor preservation, folding or blurring) and no viable tumor tissue (9%) or only very little viable tumor tissue (23%) on the slide (Figure 4B-G). Based on these findings, we provide an expert opinion on inclusion criteria of histopathological slides (Supplementary Table S5, available at https://doi.org/10.1016/j.esmoop.2022.100400).

### Diagnostic performance on biopsy samples

**Figure 5:**
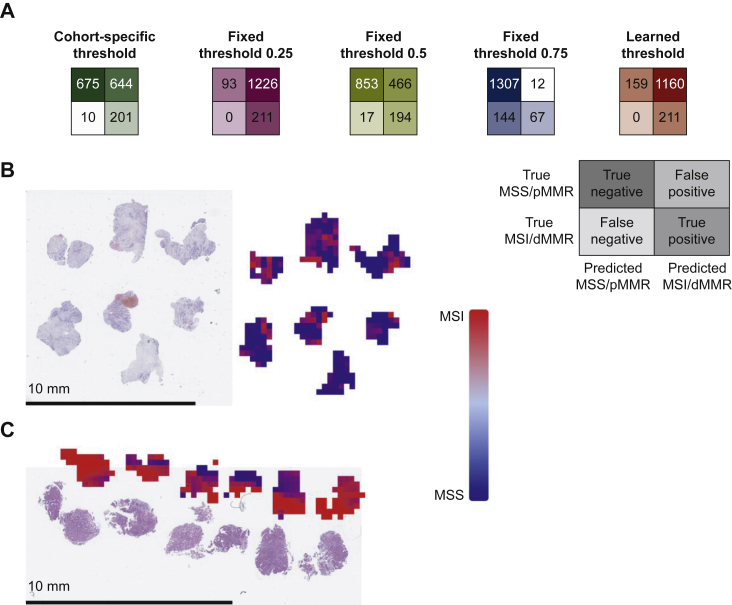
**Test performance on biopsies.** (A) Test performance based on cohort-specific threshold, fixed threshold values and learned thresholds. Confusion matrices follow the same layout as in Figure 2. (B and C) Examples of biopsy samples from the Yorkshire cohort and corresponding detection maps based on the tile-wise detection of the deep learning classifier. dMMR, mismatch repair deficiency; MSI, microsatellite instability; MSS, microsatellite stable; pMMR, proficient mismatch repair.

To evaluate the performance of the MSI/dMMR detection system in endoscopic biopsy samples, we use the classifier which was trained on all surgical samples except YCR-BCIP. We deployed this network on endoscopic biopsies from *n* = 1530 CRC patients from the Yorkshire region. We found that this system—although it was trained on surgical resection samples—yielded a high AUROC of 0.89 (0.87-0.92). When evaluated with a cohort-specific threshold that identified MSS/pMMR patients with a fixed sensitivity at 0.95, we found that 44.12% of all patients could be correctly identified as MSS/pMMR. The fixed threshold of 0.25—which had consistently achieved a good separation in surgical specimens—did not misclassify any true MSI/dMMR patients as MSS/pMMR, but at the same time it only identified 6% of all patients correctly as MSS/pMMR (Figure 5A-D). This shows that using a DL system to exclude patients from unnecessary testing is less powerful in biopsy samples than in surgical samples.

## Discussion

MSI/dMMR is a key biomarker for identification of hereditary cancer, detection of chemotherapy response and detection of immunotherapy response in CRC. Despite being recommended as a test in all CRC patients, MSI/dMMR is currently not ubiquitously tested. Previous studies have shown that DL can be used to screen for MSI/dMMR solely based on routine pathology slides^8^^,^^9^ and that implementation of this technology as a pre-screening test would lead to cost savings in health care systems.^18^ The current study aimed to quantify the percentage of patients which can be ruled out from conventional testing by an AI-based pre-screening test for MSI/dMMR in multiple populations and also provide evidence to generate practical guidelines for the optimal quality of pathology slides for AI-based diagnostic testing (Supplementary Table S5, available at https://doi.org/10.1016/j.esmoop.2022.100400). Our study shows that on average, test load in surgical specimens could be reduced by close to 50% and by 44% in biopsies when using cohort-specific thresholds. For the clinical implementation of AI systems, it is important to identify situations in which such systems fail. When AI systems are used as pre- screening tools in a cascading diagnostic workflow, FN detections are a much larger concern than FP detections. In the current study, 22 out of 8343 patients (0.26%) had FN detections when a low MSI/dMMR fixed threshold of 0.25 was used (Figure 2D). Further efforts to reduce this FN fraction seem possible by identifying specific reasons for misclassifications, although no quantitative data exist which define the level of acceptable misclassifications from the perspective of different stakeholders, including patients.

In a detailed histopathological review of these 22 cases, we identified a potential reason for this misclassification (Figure 4A): in eight (36%) of these misclassified cases, pathologists identified no tumor or only a very small portion of the tumor on the slide. In another seven (32%) of the misclassified slides, the morphology was heavily distorted by artifacts related to over-staining or tissue folds. These slides should have been—but were not—excluded before the computational image analysis which shows the importance of rigorous quality control.^37^ For another seven (32%) of the misclassified slides, no reason for misclassification could be identified. In addition, potential reasons for FP misclassification were analyzed (Figure 4B), although the level of concern about FP cases is low for a pre-screening test. Ultimately, clinical users of tests need to be aware of the avoidable and unavoidable misclassifications. To further reduce the rate of misclassifications in the future, we provide a set of expert recommendations for quality control of slides (Supplementary Table S5, available at https://doi.org/10.1016/j.esmoop.2022.100400). In addition, technical innovations could improve performance in the future, especially new models which are more robust to cohort and sample differences irrespective of the threshold values. Such generalizable models would ideally not require fine-tuning to every target population, but provide robust performance in any populations. Alternatively, however, models with imperfect generalization could be fine-tuned in specific institutions before use in diagnostic routine. Predictions of the diagnostic tests could also drift over time so repeated quality-control measures could be required in diagnostic routine. Finally, it is known that even the gold-standard tests for MSI/dMMR do not have 100% sensitivity and specificity.^38^ Therefore, it is conceivable that some ‘misclassified’ cases actually represent misclassifications by the ground truth method.

In this study, most cohorts yielded consistent classification results, including a cohort of patients with CRC and inflammatory bowel diseases (‘UMM’ cohort, Figure 1B), constituting a rare but clinically relevant subgroup. However, a lower performance was observed in samples from the MECC study (Figure 2). One potential contributing factor to this lower performance is the high percentage of MSI cases in this cohort. To find additional reasons for the comparably low performance in this cohort, we reviewed the histopathological quality of the scanned slides as well as the technical specifications of the digitized data files but did not identify relevant differences compared to the other patient cohorts. However, a possible reason for the overall lower performance of the system in samples from the MECC study is the ethnicity of the patient population included in this study: This study is a population-based study from northern Israel and has a high proportion of Ashkenazi Jews who have a specific genetic mechanism in familial CRC^39^ and potentially differences in genetics of sporadic CRC, as evident by a higher proportion of BRAF mutations in CRC.^40^ These ethnic differences could conceivably result in a lower performance. For future studies, it is therefore important to record and specifically analyze the performance of AI-based diagnostic systems with respect to ethnicity. Except for ethnicity, other clinicopathological features did not show any obvious interrelation with classification performance (Supplementary Table S3, available at https://doi.org/10.1016/j.esmoop.2022.100400).^10^

Additional limitations of our study are that our AI system is a non-clinically approved research tool. Based on our results, MSI/dMMR detection systems could and should be built as a diagnostic device with regulatory approval. In addition, although our method addressed cohort-specific thresholds, other ways of addressing a distribution shift in different populations could be investigated by future studies, for instance improved data normalization procedures.

Taken together, these data quantify potential resource savings that could be achieved by DL-based MSI/dMMR testing in diagnostic routine in multiple independent patient cohorts. Our findings provide a quantitative benchmark for future technological improvements and evaluation of the system in diagnostic routine. In addition, by identifying potential reasons for misclassifications in the underlying technology, our study provides heuristics (Supplementary Table S5, available at https://doi.org/10.1016/j.esmoop.2022.100400) of potential practical utility for future applications of AI-based diagnostic systems in histopathology.
