# Supplementary material for: Artificial intelligence for detection of microsatellite instability in colorectal cancer—a multicentric analysis of a pre-screening tool for clinical application
Source: ESMO Open. 2022 Mar 2;7(2):100400. doi: 10.1016/j.esmoop.2022.100400 (PMC9058894; doi:10.1016/j.esmoop.2022.100400)
Supplement: Supplementary Figures S1-14 [file mmc1.docx]

# Supplementary Figures

**
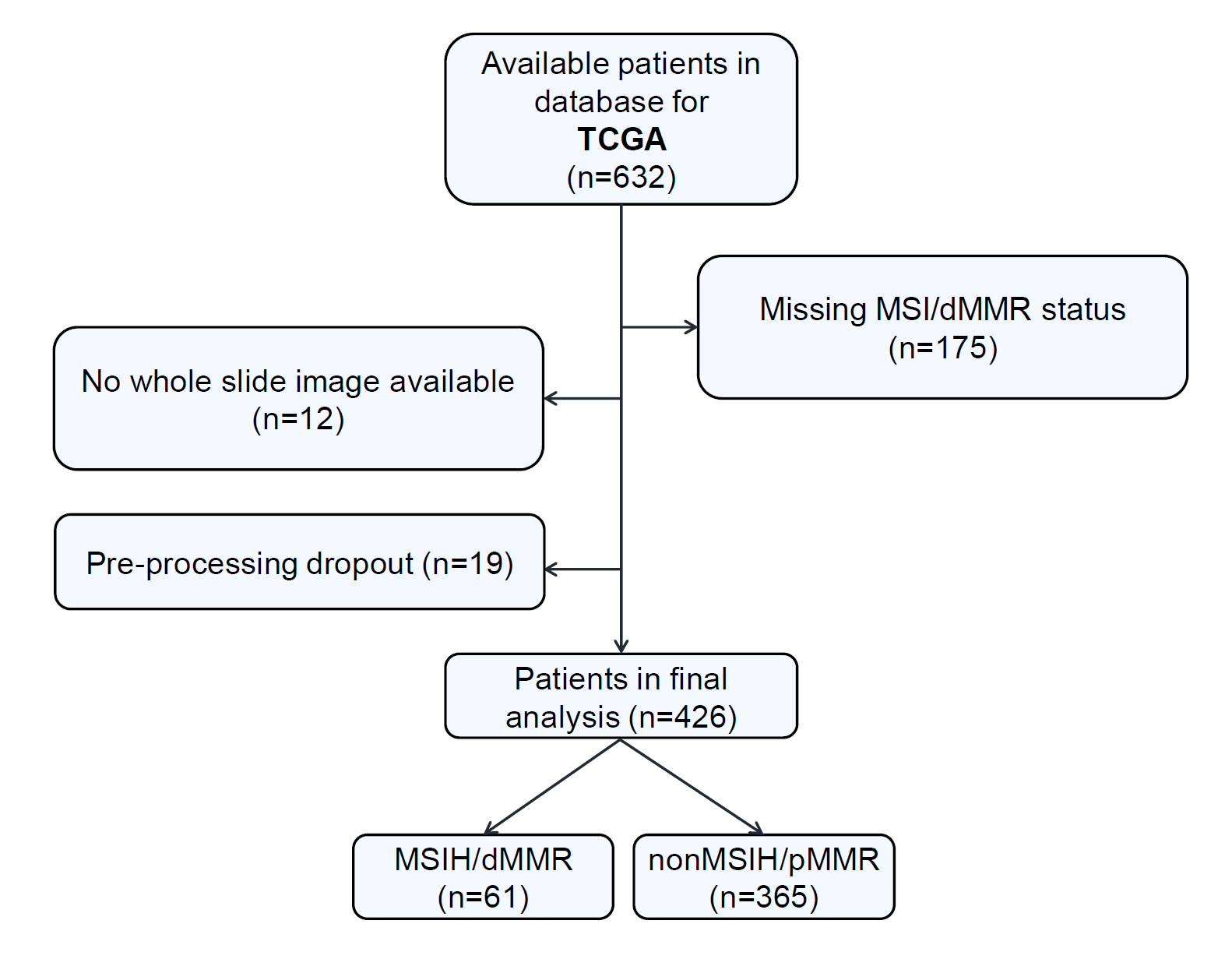
**

**Suppl. Figure 1: CONSORT chart for cohort TCGA**

**
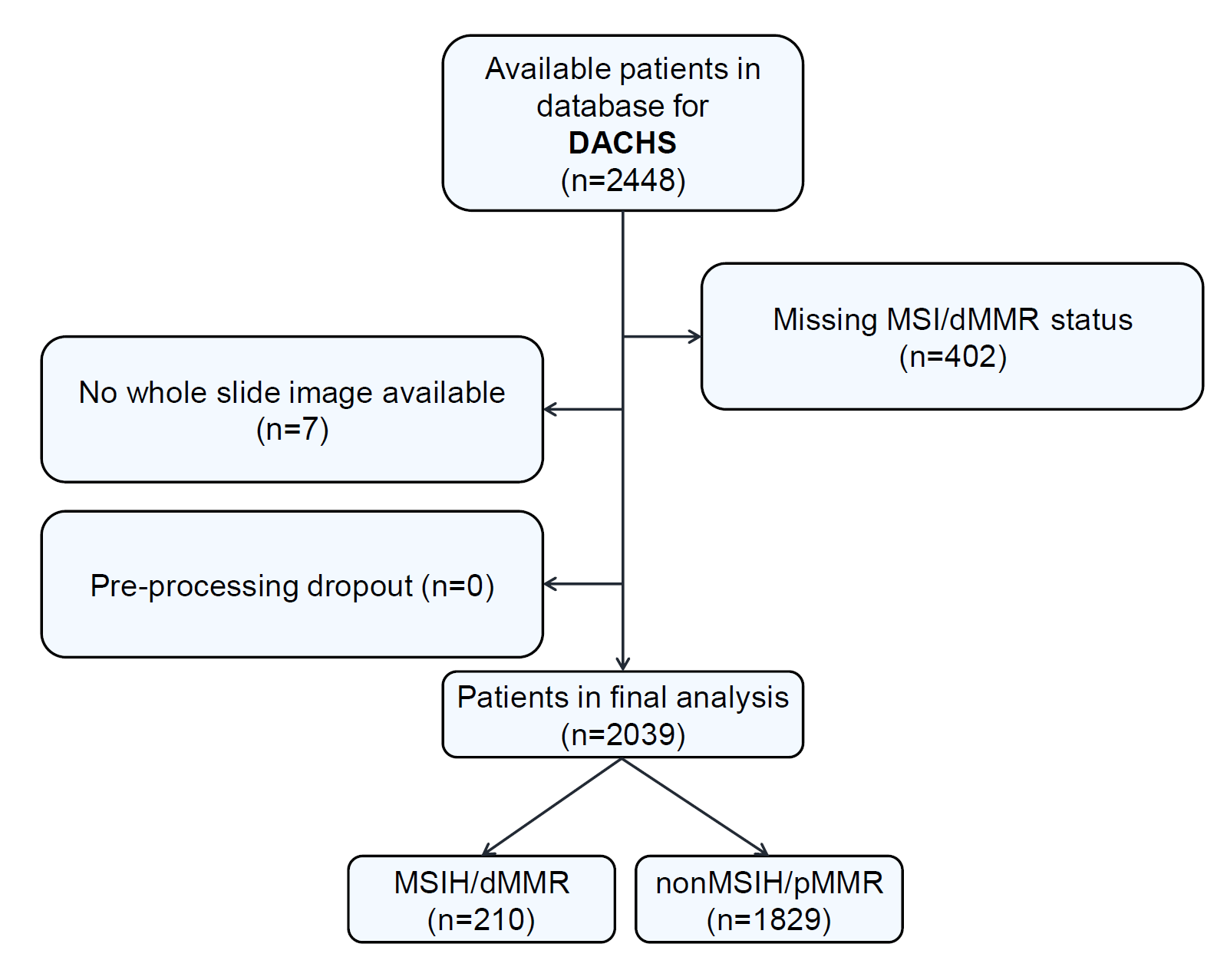
**

**Suppl. Figure 2: CONSORT chart for cohort DACHS**

**
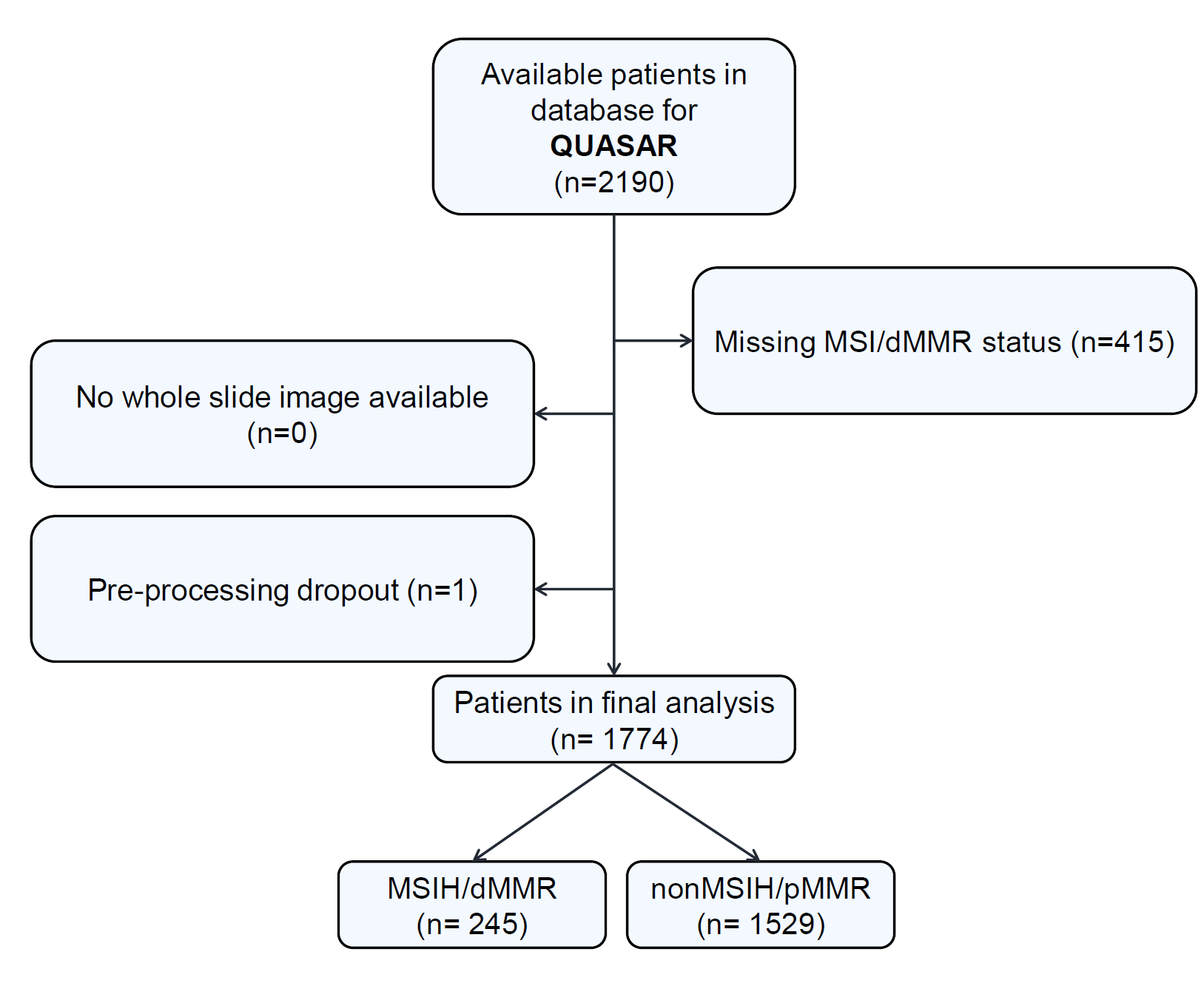
**

**Suppl. Figure 3: CONSORT chart for cohort QUASAR.**

**
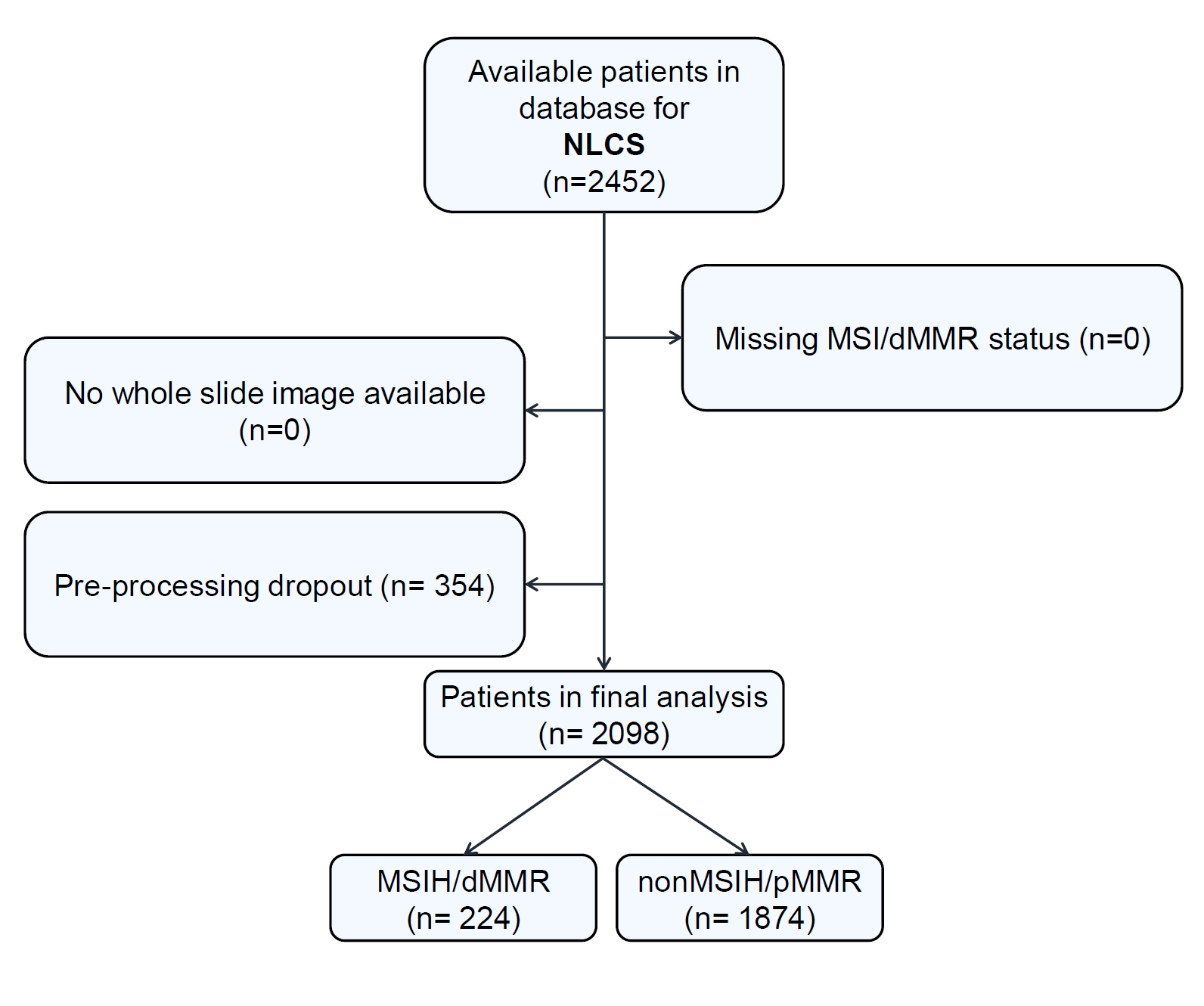
**

**Suppl. Figure 4: CONSORT chart for cohort NLCS.**

**
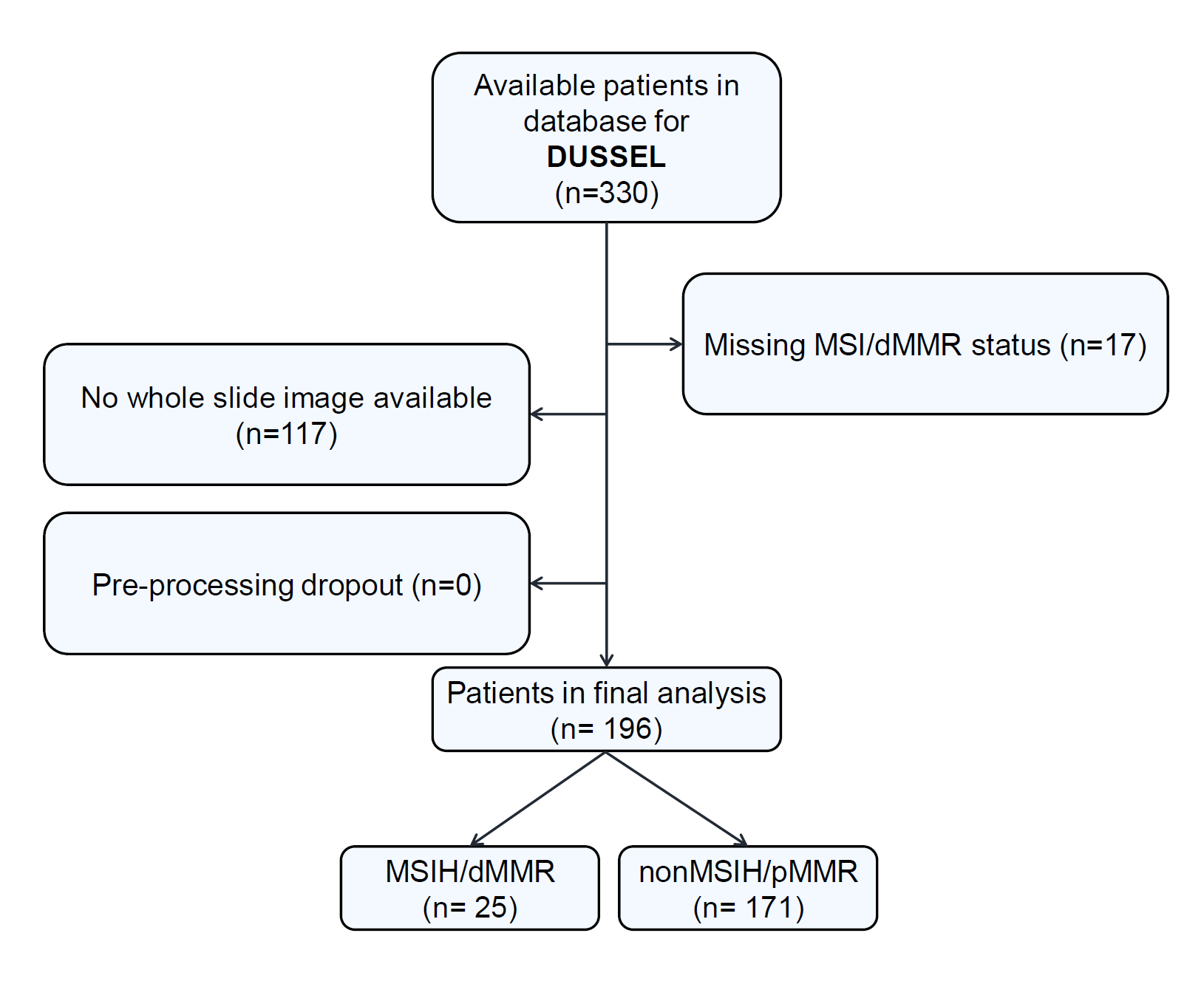
**

**Suppl. Figure 5: CONSORT chart for cohort DUSSEL.**

**
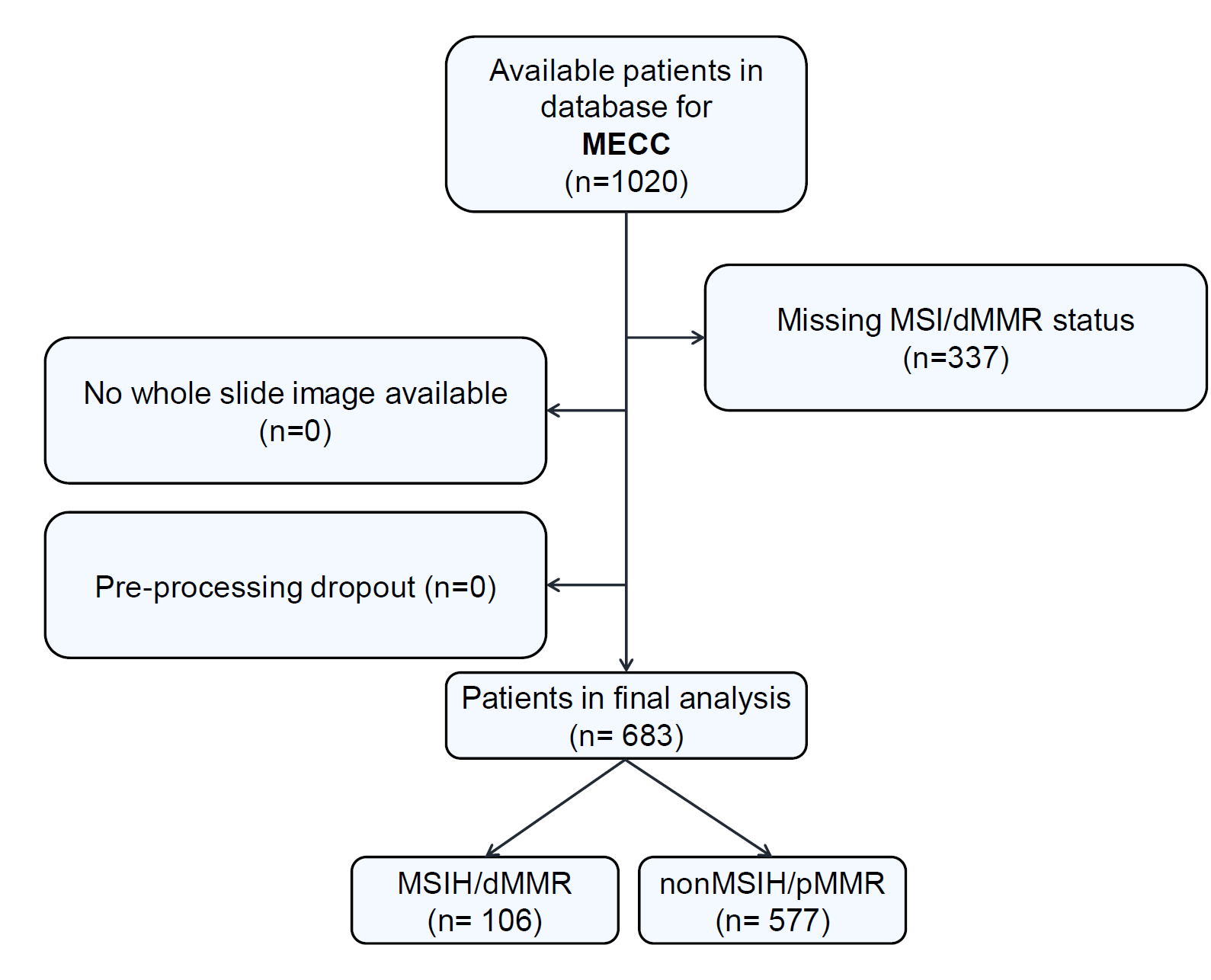
**

**Suppl. Figure 6: CONSORT chart for cohort MECC**

**
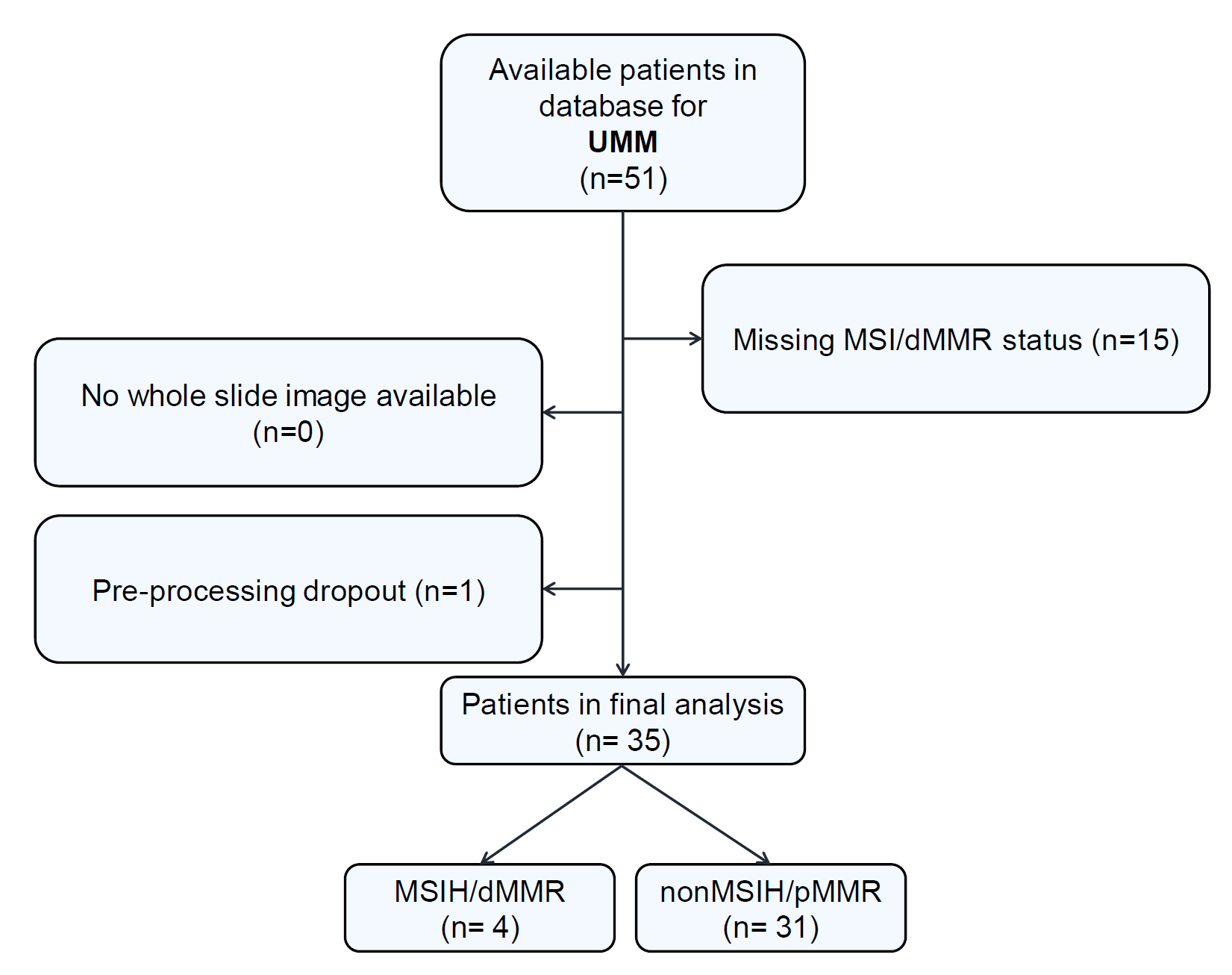
**

**Suppl. Figure 7: CONSORT chart for cohort UMM.**

**
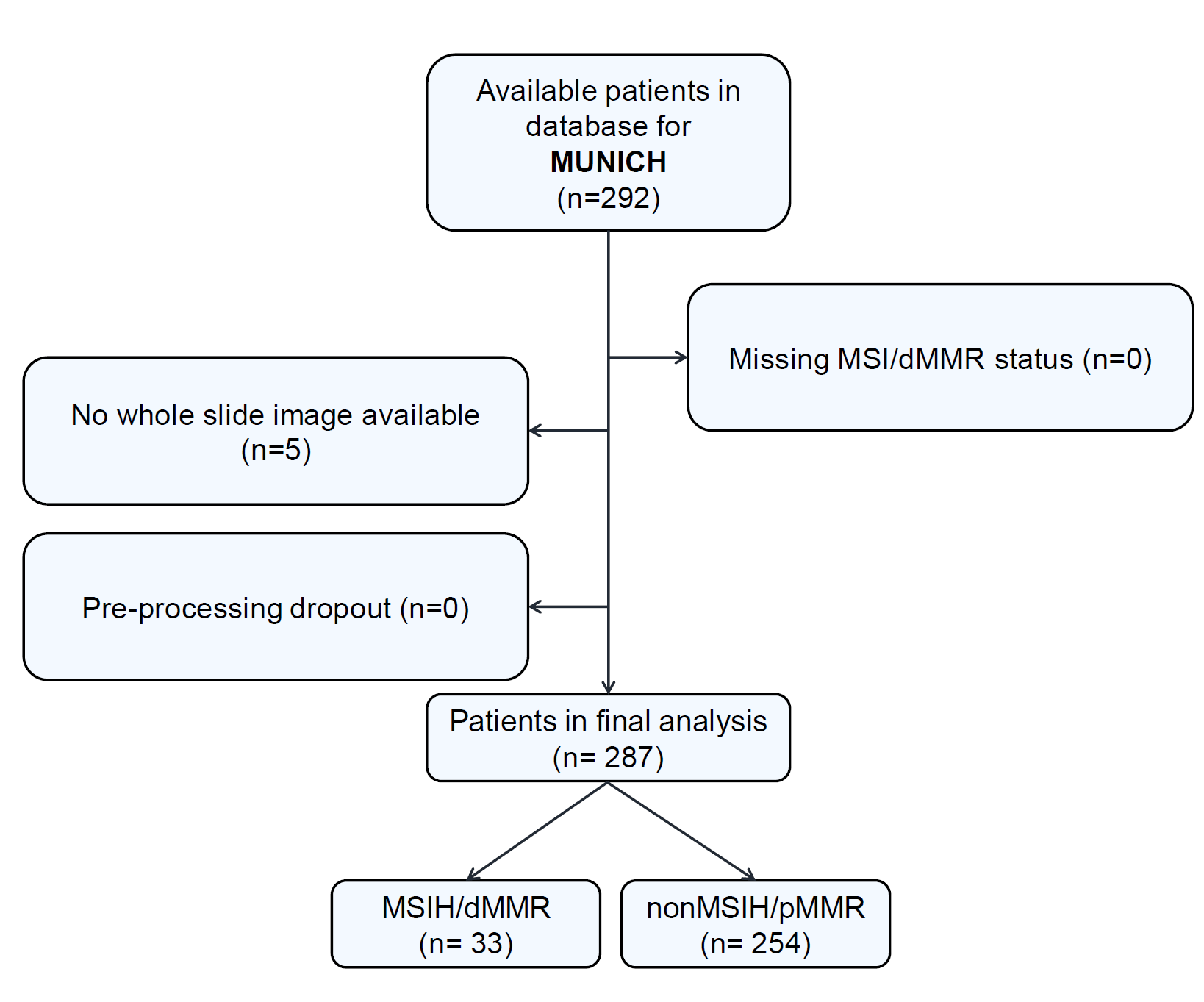
**

**Suppl. Figure 8: CONSORT chart for cohort MUNICH.**

**
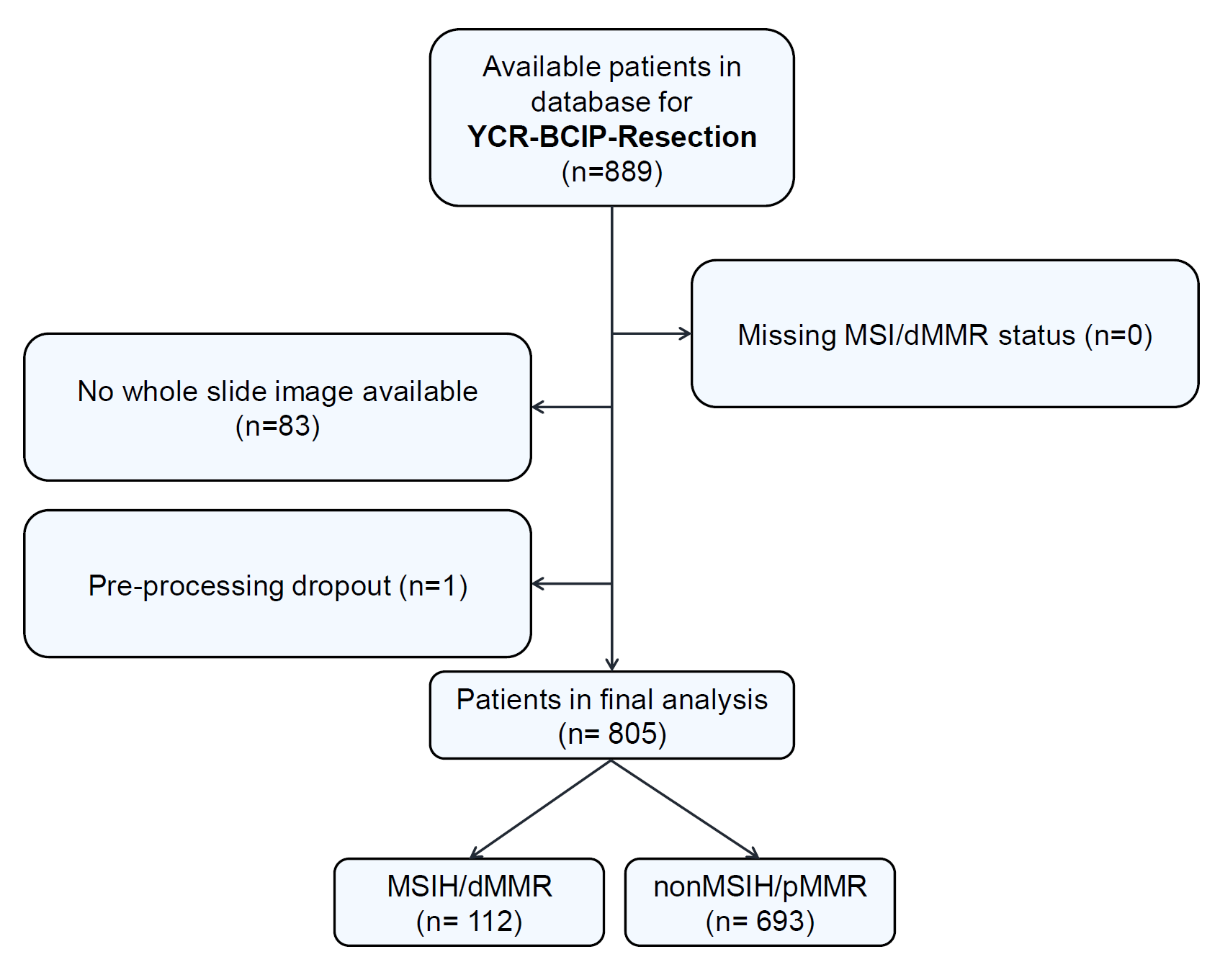
**

**Suppl. Figure 9: CONSORT chart for cohort YCR-BCIP-Resection.**

**
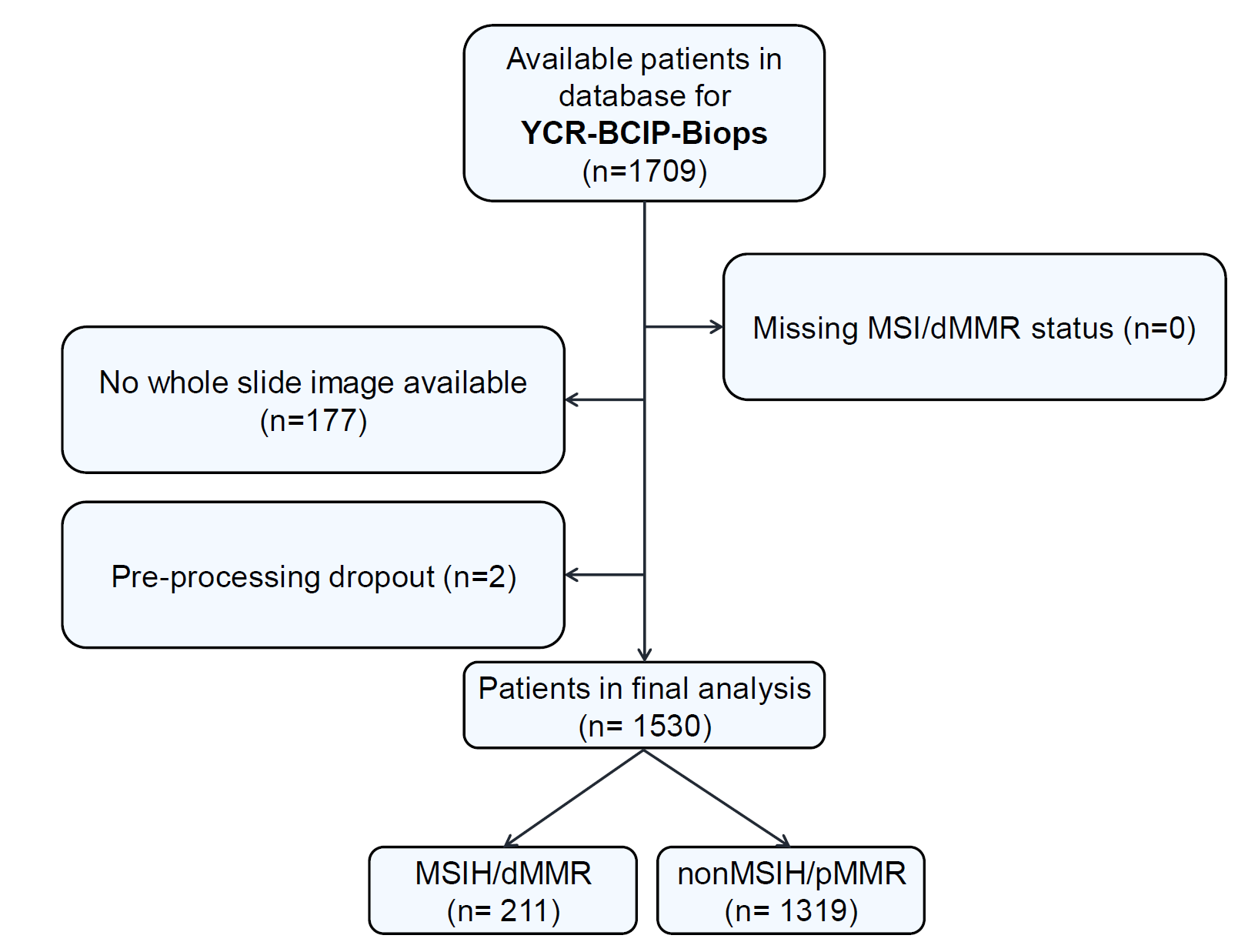
**

**Suppl. Figure 10: CONSORT chart for cohort YCR-BCIP-Biopsies.**

#


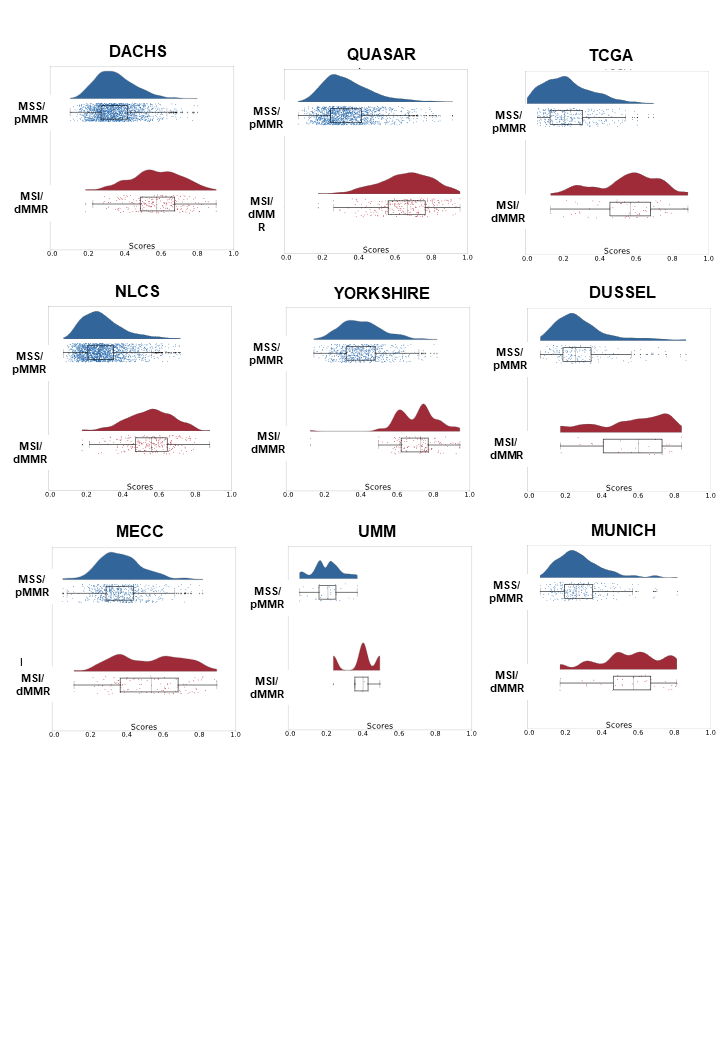


**Suppl. Figure 11: Distribution of the Deep Learning scores for MSS/pMMR patients (blue) and MSI/dMMR patients (red) in all cohorts.** A perfect classifier will yield two non-overlapping distributions. A random classifier will yield two completely overlapping distributions.


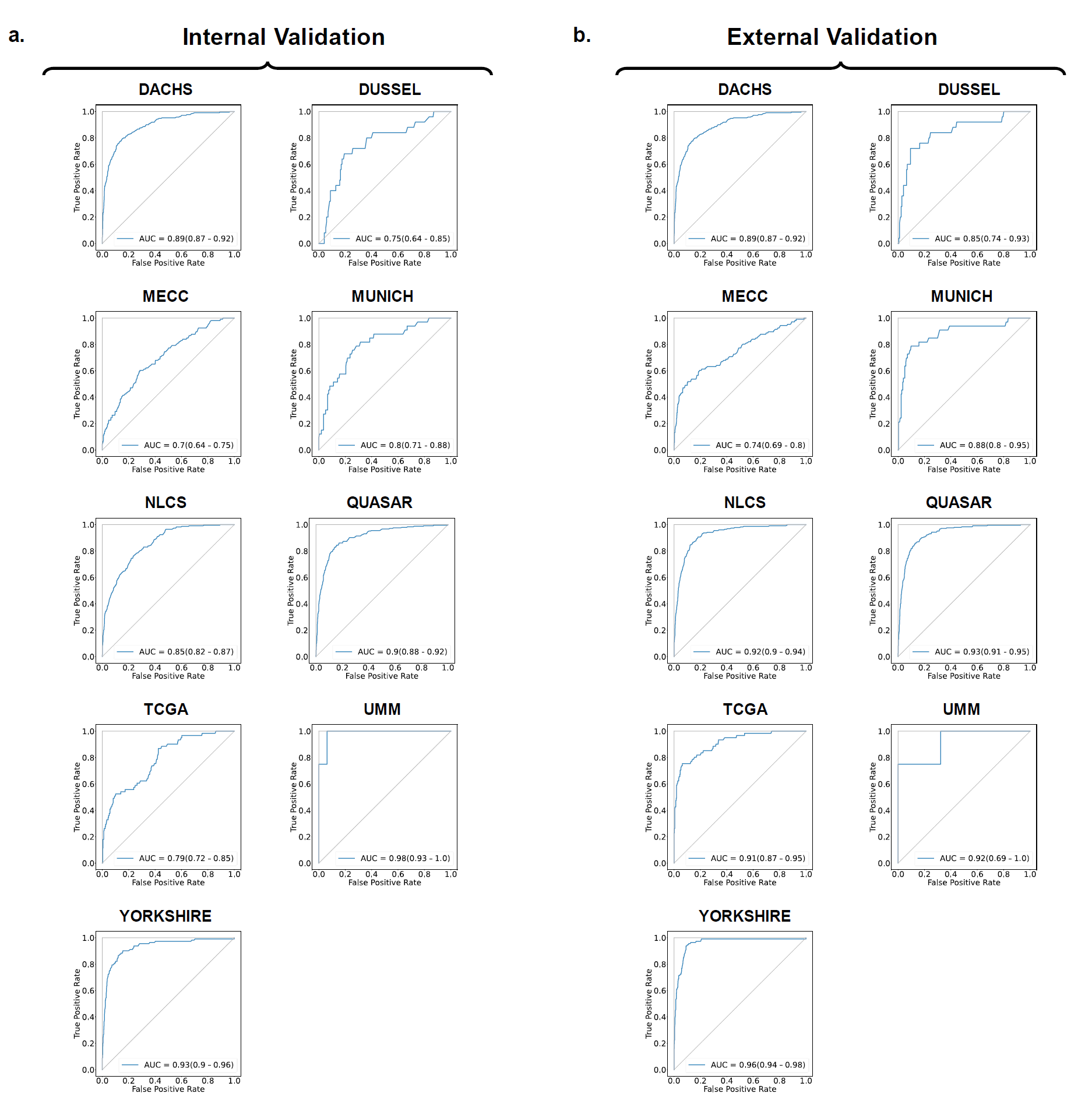


**Suppl. Figure 12: Receiver Operating Characteristic (ROC) curves for internal cross-validation and external validation experiments.**
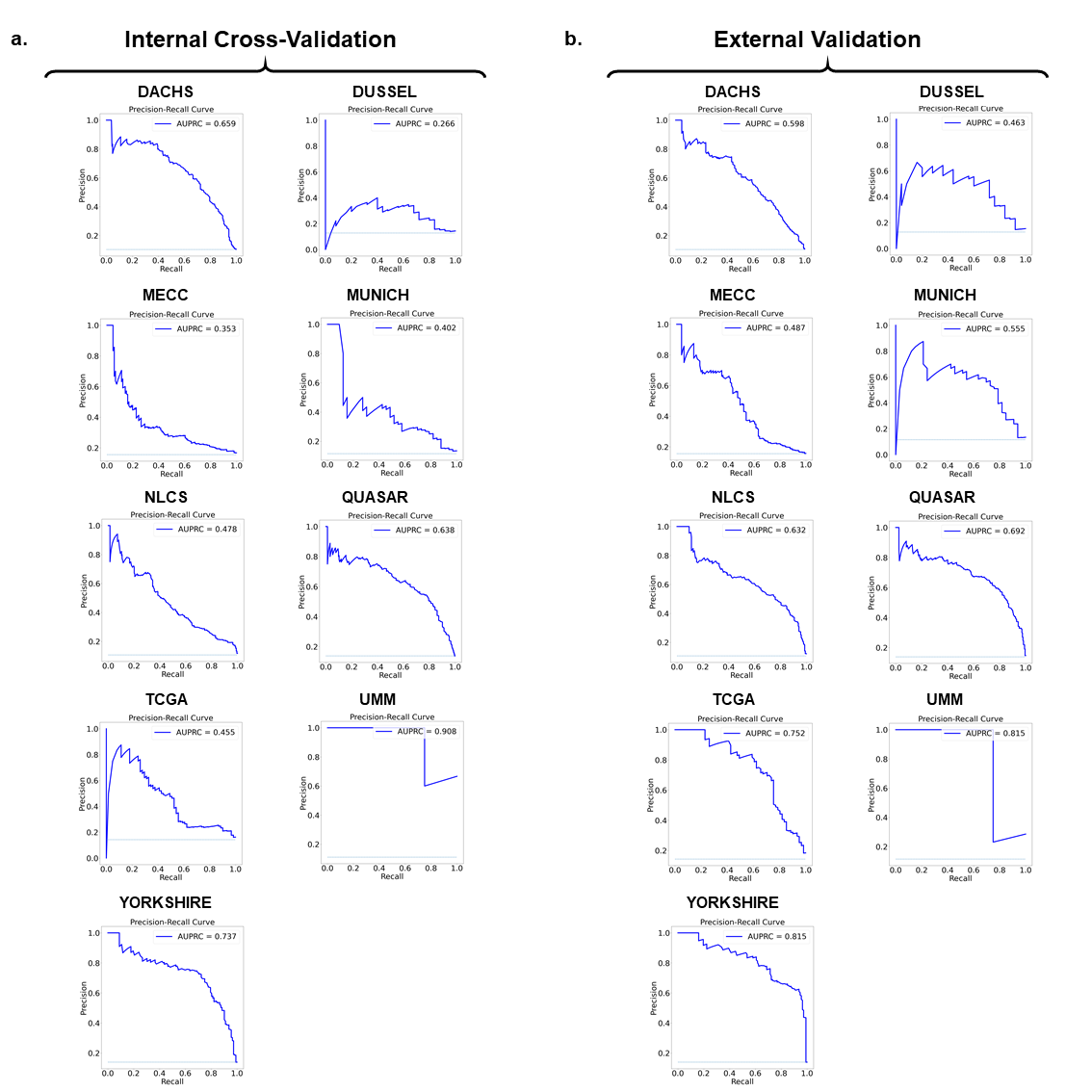


**Suppl. Figure 13: Precision-Recall curves for internal cross-validation and external validation experiments.**


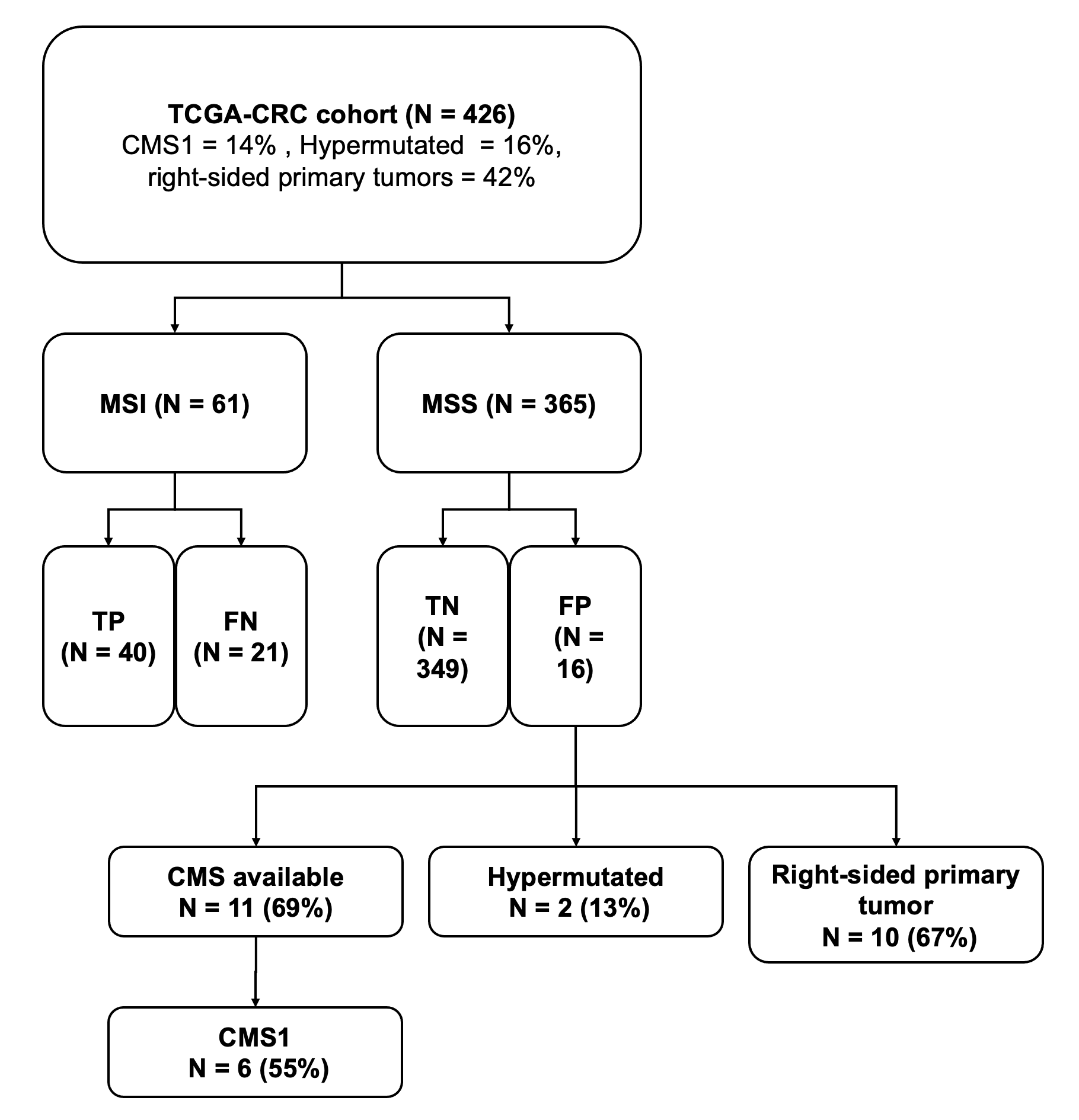


**Suppl. Figure 14: Characteristics of misclassified cases in the TCGA cohort.** MSI = microsatellite unstable, MSS = microsatellite stable, TP = true positive, FN = false negative, TN = true negative, FP = false positive, CMS = consensus molecular subtype according to [[35]](https://paperpile.com/c/cXUhwy/2fQyb).
