# Supplementary material for: Artificial intelligence for detection of microsatellite instability in colorectal cancer—a multicentric analysis of a pre-screening tool for clinical application
Source: ESMO Open. 2022 Mar 2;7(2):100400. doi: 10.1016/j.esmoop.2022.100400 (PMC9058894; doi:10.1016/j.esmoop.2022.100400)
Supplement: Supplementary Tables S1-S6 [file mmc2.docx]

# Supplementary Tables

| **Cohort** | **Ethics approval ID** | **Ethics board** | **Patient consent statement** |
| --- | --- | --- | --- |
| DACHS[[41]](https://paperpile.com/c/cXUhwy/5xTJA) | 310/2001 | Ethics committee of the Medical Faculty, University of Heidelberg (Erstvotum) | Informed consent |
| QUASAR[[24]](https://paperpile.com/c/cXUhwy/UJZru) | 08/H0903/62 | North East – York Research Ethics Committee | Informed consent |
| TCGA[[25]](https://paperpile.com/c/cXUhwy/emqbm) | Not applicable | Not applicable | Informed consent |
| NLCS[[42–44]](https://paperpile.com/c/cXUhwy/a28w8+1wOCv+43RaR) | METC number 85-012 | Approved by the institutional review boards of the TNO Quality of Life Research Institute (Zeist, NL) and Maastricht University (Maastricht, NL). Ethical approval was obtained from the Medical Ethical Committee (METC) of Maastricht University Medical Center+. | Informed consent |
| YCR-BCIP[[4]](https://paperpile.com/c/cXUhwy/6pkdt) | Not applicable | Ethical approval was not required, because DG27 was already in place recommending LS screening in all patients diagnosed with CRC. Testing was considered part of the ‘standard of care’ clinical pathway. | Specific patient consent not obtained |
| DUSSEL[[30]](https://paperpile.com/c/cXUhwy/fmWC0) | Not applicable | Local ethics regulations at the time of sample collection | Specific patient consent not required |
| MECC[[21]](https://paperpile.com/c/cXUhwy/r7FcN) | IRB #19404 | City of Hope National Medical Center, Duarte, CA and local IRB CHS National Israeli Cancer Control Center | Informed consent |
| UMM | 2015-815R-MA | Medical Ethics Commission II of the Medical Faculty Mannheim, Heidelberg University, Mannheim, Germany | Specific patient consent not required |
| MUNICH | 2136/08 | local ethics committee of Technical University Munich | Informed consent |

**Suppl. Table 1: Ethics statement of the selected cohorts for this study. NL: The Netherlands.**

#

| Section & Topic | No | Item | Reported |
| --- | --- | --- | --- |
| TITLE OR ABSTRACT | 1 | Identification as a study of diagnostic accuracy using at least one measure of accuracy (such as sensitivity, specificity, predictive values, or AUC) | yes |
| ABSTRACT | 2 | Structured summary of study design, methods, results, and conclusions (for specific guidance, see STARD for Abstracts) | yes |
| INTRODUCTION | 3 | Scientific and clinical background, including the intended use and clinical role of the index test | yes |
|  | 4 | Study objectives and hypotheses | yes |
| METHODS  Study design | 5 | Whether data collection was planned before the index test and reference standard were performed (prospective study) or after (retrospective study) |  |
| METHODS  Participants | 6 | Eligibility criteria |  |
|  | 7 | On what basis potentially eligible participants were identified (such as symptoms, results from previous tests, inclusion in registry) |  |
|  | 8 | Where and when potentially eligible participants were identified (setting, location and dates) |  |
|  | 9 | Whether participants formed a consecutive, random or convenience series |  |
| METHODS  Test methods | 10a | Index test, in sufficient detail to allow replication | yes |
|  | 10b | Reference standard, in sufficient detail to allow replication | yes |
|  | 11 | Rationale for choosing the reference standard (if alternatives exist) |  |
|  | 12a | Definition of and rationale for test positivity cut-offs or result categories of the index test, distinguishing pre-specified from exploratory | yes |
|  | 12b | Definition of and rationale for test positivity cut-offs or result categories of the reference standard, distinguishing pre-specified from exploratory |  |
|  | 13a | Whether clinical information and reference standard results were available to the performers/readers of the index test |  |
|  | 13b | Whether clinical information and index test results were available to the assessors of the reference standard |  |
| METHODS  Analysis | 14 | Methods for estimating or comparing measures of diagnostic accuracy | yes |
|  | 15 | How indeterminate index test or reference standard results were handled |  |
|  | 16 | How missing data on the index test and reference standard were handled | yes |
|  | 17 | Any analyses of variability in diagnostic accuracy, distinguishing pre-specified from exploratory |  |
|  | 18 | Intended sample size and how it was determined | yes |
| RESULTS  Participants | 19 | Flow of participants, using a diagram | yes |
|  | 20 | Baseline demographic and clinical characteristics of participants |  |
|  | 21a | Distribution of severity of disease in those with the target condition |  |
|  | 21b | Distribution of alternative diagnoses in those without the target condition | yes |
|  | 22 | Time interval and any clinical interventions between index test and reference standard |  |
| RESULTS  Test results | 23 | Cross tabulation of the index test results (or their distribution) by the results of the reference standard |  |
|  | 24 | Estimates of diagnostic accuracy and their precision (such as 95% confidence intervals) | yes |
|  | 25 | Any adverse events from performing the index test or the reference standard |  |
| DISCUSSION | 26 | Study limitations, including sources of potential bias, statistical uncertainty, and generalisability | yes |
|  | 27 | Implications for practice, including the intended use and clinical role of the index test |  |
| OTHER INFORMATION | 28 | Registration number and name of registry |  |
|  | 29 | Where the full study protocol can be accessed |  |
|  | 30 | Sources of funding and other support; role of funders | yes |

**Suppl. Table 2: STARD (STAndards for the Reporting of Diagnostic accuracy studies) Checklist.**

|  | **DACHS** | **QUASAR** | **TCGA** | **NLCS** | **YCR-BCIP** | **DUSSEL** | **MECC**** | **UMM** | **MUNICH** |
| --- | --- | --- | --- | --- | --- | --- | --- | --- | --- |
| **Origin** | Germany | United Kingdom | United States | The Netherlands | United Kingdom | Germany | Israel | Germany | Germany |
| **Number of patients*** | 2448 | 2190 | 632 | 2452 | 889 | 330 | 683 | 51 | 292 |
| **WSI format** | SVS | SVS | SVS | TIFF/SVS | SVS | SVS | TIF | CZI | SVS |
| **MSI/dMMR ground truth** | PCR 3-plex | IHC 4-plex or IHC 2-plex | PCR 5-plex [[36]](https://paperpile.com/c/cXUhwy/l2LAT) | IHC 2-plex | IHC 4-plex | IHC 2-plex | PCR 5-plex | 4 PCR  20 IHC  11 both | IHC 4-plex |
| **MSI/dMMR, n (%)** | 210  (9%) | 246  (11%) | 65  (10%) | 259  (11%) | 129  (15%) | 45  (14%) | 106  (16%) | 4  (8%) | 34  (12%) |
| **MSS/pMMR, n(%)** | 1836  (75%) | 1529  (70%) | 392  (62%) | 2193  (89%) | 760  (85%) | 268  (81%) | 577  (84%) | 32  (63%) | 258  (88%) |
| **Mean age at diagnosis (standard deviation)** | 68.46  ($\pm$10.82) | 62.20  ($\pm$9.60) | 66.42  ($\pm$12.67) | 73.71  ($\pm$6.04) | 70.31  ($\pm$9.97) | 68.57  ($\pm$11.77) | 69.8 | 54.42  ($\pm$11.28) | 56.1  ($\pm$11.84) |
| **Colon cancer, n (%)** | 1488  (61%) | 1474  (67%) | 341  (54%) | 1730  (71%) | 667  (75%) | 204  (62%) | 530  (78%) | 23  (45%) | N/A |
| **Rectal cancer, n (%)** | 960  (39%) | 526  (24%) | 118  (19%) | 722  (29%) | 215  (24%) | 116  (35%) | 123  (18%) | 22  (43%) | N/A |
| **Organ unknown, n(%)** | 0  (0%) | 190  (9%) | 173  (27%) | 0  (0%) | 7  (1%) | 10  (3%) | 30  (4%) | 6  (12%) | N/A |
| **Female,**  **n (%)** | 1012  (41%) | 848  (39%) | 292  (46%) | 1079  (44%) | 395  (44%) | 181  (55%) | 320  (47%) | 27  (53%) | 132  (45%) |
| **Male,**  **n (%)** | 1436  (59%) | 1334  (61%) | 322  (51%) | 1373  (56%) | 494  (56%) | 149  (45%) | 363  (53%) | 24  (47%) | 160  (55%) |
| **gender unknown** | 0  (0%) | 8  (0%) | 18  (3%) | 0  (0%) | 0  (0%) | 0  (0%) | 0  (0%) | 0  (0%) | 0 (0%) |
| **UICC stage I, n (%)** | 485  (20%) | 1  (0%) | 76  (12%) | 485  (20%) | 169  (19%) | 76  (23%) | 94  (14%) | 6  (12%) | 52  (18%) |
| **UICC stage II, n (%)** | 801  (33%) | 1988  (91%) | 166  (26%) | 918  (37%) | 317  (36%) | 138  (42%) | 335  (49%) | 24  (47%) | 118  (40%) |
| **UICC stage III, n (%)** | 822  (34%) | 192  (9%) | 140  (22%) | 641  (26%) | 370  (42%) | 110  (33%) | 123  (18%) | 9  (18%) | 82  (28%) |
| **UICC stage IV, n (%)** | 337  (14%) | 0  (0%) | 63  (10%) | 341  (14%) | 0  (0%) | 6  (2%) | 67  (10%) | 5  (10%) | 39  (13%) |
| **UICC stage unknown** | 3  (0%) | 9  (0%) | 187  (30%) | 67  (3%) | 33  (3%) | 0  (0%) | 64  (9%) | 7  (13%) | 1  (0%) |
| **BRAF mutation, n (%)** | 151  (6%) | 120  (5%) | 63  (10%) | N/A | 75  (8%) | N/A | 49  (7%) | 2  (4%) | N/A |
| **BRAF wild type, n(%)** | 1930  (79%) | 1358  (62%) | 471  (75%) | N/A | 32  (4%) | N/A | 570  (83%) | 7  (14%) | N/A |
| **BRAF status unknown** | 367  (15%) | 712  (33%) | 98  (15%) | N/A | 782  (88%) | N/A | 64  (9%) | 42  (82%) | N/A |
| **KRAS mutation, n (%)** | 677  (28%) | 555  (25%) | 218  (34%) | N/A | N/A | N/A | 252  (37%) | N/A | N/A |
| **KRAS wild type, n (%)** | 1397  (57%) | 882  (40%) | 316  (50%) | N/A | N/A | N/A | 405  (59%) | N/A | N/A |
| **KRAS status unknown** | 347  (15%) | 753  (35%) | 98  (16%) | N/A | N/A | N/A | 26  (4%) | N/A | N/A |
| **right-sided tumor, n (%)** | 819  (33%) | 754  (34%) | 176  (28%) | 946  (39%) | 331  (37%) | 72  (22%) | 238  (35%) | 22  (43%) | 53  (18%) |
| **left-sided tumor, n (%)** | 1607  (66%) | 1158  (53%) | 248  (39%) | 1506  (61%) | 486  (55%) | 226  (68%) | 409  (60%) | 24  (47%) | 239  (82%) |
| **sidedness unknown, n(%)** | 22  (1%) | 150  (13%) | 208  (33%) | 0 (0%) | 72  (8%) | 32  (10%) | 36  (5%) | 5  (10%) | 0 (0%) |
| **etiology** | any | not specified | not specified | not specified | not specified | not specified | not specified | IBD | not specified |

**Suppl. Table 3: : Patient cohorts used in this study and their characteristics.** Clinico-pathological data were provided by the respective study principal investigators. In all cases, the TNM version from the original study registry was used. Information about the localization of the tumour was either provided as a binary variable (left-sided vs. right-sided) by the study site or assigned by the authors as follows: the cecum, ascending colon, hepatic flexure and transverse colon were defined as a right-sided tumour location whereas the splenic flexure, descending colon, sigmoid colon and rectum were defined as left-sided. * Number of patients before dropout of samples. For detailed information see Suppl. Figures 1-10. ** for the MECC cohort, these statistics refer to the cases with available MSI/dMMR status only.

|  | **Cohort Specific Threshold** | **Learned Threshold** |
| --- | --- | --- |
| **DACHS** | 0.338 | 0.293 |
| **DUSSEL** | 0.188 | 0.304 |
| **MECC** | 0.248 | 0.300 |
| **MUNICH** | 0.236 | 0.305 |
| **QUASAR** | 0.398 | 0.292 |
| **NLCS** | 0.328 | 0.293 |
| **TCGA** | 0.220 | 0.297 |
| **UMM** | 0.244 | 0.257 |
| **YCR-BCIP** | 0.568 | 0.289 |
| **YCR-BCIP-Biopsies** | 0.446 | 0.289 |

**Suppl. Table 4: Cohort Specific and Learned Thresholds.**  Cohort specific threshold is calculated for each individual test cohort. Learned threshold is obtained by averaging the optimal thresholds of all 8 training cohorts which is calculated at fixed sensitivity of 95%.

|  | **Recommendation** | **Justification** |
| --- | --- | --- |
| #1 | No artifacts should be present on slide (such as blurriness, tissue folds or air bubbles). | Among the misclassified cases in our study, artifacts were commonly observed. |
| #2 | at least 20% of the tissue on the slide should be tumor | A low tumor percentage might lead to false negative detection results as we identified when reviewing misclassified cases. |
| #3 | Only formalin-fixed paraffin-embedded (FFPE) material should be used | the AI system is trained on FFPE material and the performance on frozen sections is unclear |
| #4 | No pen marks should be present on the slides | Although in this study, we had no indication that adversely affected the performance, this should be avoided for consistency. |
| #5 | Largely mucinous tumors should be validated with a gold standard method. | Among the misclassified cases, we identified some tissue slides which had mostly (>50%) mucin on the slide. This amorphous mass can potentially lead to false positive misclassifications. |
| #6 | Largely necrotic tumors should be validated with a gold standard method. | Similarly to #5, we identified some tissue slides which had mostly (>50%) necrotic tissue on the slide. This acellular mass can potentially lead to misclassifications. |

**Suppl. Table 5: Expert opinion on optimal inclusion criteria of histopathological slides in AI-based diagnostic tests for MSI/dMMR.**

| Rainbow-TMA project group | P.A. van den Brandt, A. zur Hausen, H. Grabsch, M. van Engeland, L.J. Schouten, J. Beckervordersandforth (Maastricht University Medical Center, Maastricht, Netherlands); P.H.M. Peeters, P.J. van Diest, H.B. Bueno de Mesquita (University Medical Center Utrecht, Utrecht, Netherlands); J. van Krieken, I. Nagtegaal, B. Siebers, B. Kiemeney (Radboud University Medical Center, Nijmegen, Netherlands); F.J. van Kemenade, C. Steegers, D. Boomsma, G.A. Meijer (VU University Medical Center, Amsterdam, Netherlands); F.J. van Kemenade, B. Stricker (Erasmus University Medical Center, Rotterdam, Netherlands); L. Overbeek, A. Gijsbers (PALGA, the Nationwide Histopathology and Cytopathology Data Network and Archive, Houten, Netherlands) |
| --- | --- |
| Rainbow-TMA collaborating pathologists, among others | A. de Bruïne (VieCuri Medical Center, Venlo); J.C. Beckervordersandforth (Maastricht University Medical Center, Maastricht); J. van Krieken, I. Nagtegaal (Radboud University Medical Center, Nijmegen); W. Timens (University Medical Center Groningen, Groningen); F.J. van Kemenade (Erasmus University Medical Center, Rotterdam); M.C.H. Hogenes (Laboratory for Pathology Oost-Nederland, Hengelo); P.J. van Diest (University Medical Center Utrecht, Utrecht); R.E. Kibbelaar (Pathology Friesland, Leeuwarden); A.F. Hamel (Stichting Samenwerkende Ziekenhuizen Oost-Groningen, Winschoten); A.T.M.G. Tiebosch (Martini Hospital, Groningen); C. Meijers (Reinier de Graaf Gasthuis/ S.S.D.Z., Delft); R. Natté (Haga Hospital Leyenburg, The Hague); G.A. Meijer (VU University Medical Center, Amsterdam); J.J.T.H. Roelofs (Academic Medical Center, Amsterdam); R.F. Hoedemaeker (Pathology Laboratory Pathan, Rotterdam); S. Sastrowijoto (Orbis Medical Center, Sittard); M. Nap (Atrium Medical Center, Heerlen); H.T. Shirango (Deventer Hospital, Deventer); H. Doornewaard (Gelre Hospital, Apeldoorn); J.E. Boers (Isala Hospital, Zwolle); J.C. van der Linden (Jeroen Bosch Hospital, Den Bosch); G. Burger (Symbiant Pathology Center, Alkmaar); R.W. Rouse (Meander Medical Center, Amersfoort); P.C. de Bruin (St. Antonius Hospital, Nieuwegein); P. Drillenburg (Onze Lieve Vrouwe Gasthuis, Amsterdam); C. van Krimpen (Kennemer Gasthuis, Haarlem); J.F. Graadt van Roggen (Diaconessenhuis, Leiden); S.A.J. Loyson (Bronovo Hospital, The Hague); J.D. Rupa (Laurentius Hospital, Roermond); H. Kliffen (Maasstad Hospital, Rotterdam); H.M. Hazelbag (Medical Center Haaglanden, The Hague); K. Schelfout (Stichting Pathologisch en Cytologisch Laboratorium West-Brabant, Bergen op Zoom); J. Stavast (Laboratorium Klinische Pathologie Centraal Brabant, Tilburg); I. van Lijjnschoten (PAMM Laboratory for Pathology and Medical Microbiology, Eindhoven); K. Duthoi (Amphia Hospital, Breda) |

**Suppl. Table 6**: The Rainbow-TMA Consortium contributing samples for the NLCS study.

# References for supplementary tables

The numbering of these references corresponds to the numbering of references in the main manuscript. All references which (re)appear in the Suppl. Tables are listed here.

4. West NP, Gallop N, Kaye D et al. Lynch syndrome screening in colorectal cancer: results of a prospective two-year regional programme validating the NICE diagnostics guidance pathway across a 5.2 million population. Histopathology 2021. doi:10.1111/his.14390.

21. Hoffmeister M, Jansen L, Rudolph A et al. Statin use and survival after colorectal cancer: the importance of comprehensive confounder adjustment. J. Natl. Cancer Inst. 2015; 107(6):djv045.

24. QUASAR Collaborative Group. Adjuvant chemotherapy versus observation in patients with colorectal cancer: a randomised study. Lancet 2007; 370(9604):2020–2029.

25. Cancer Genome Atlas Network. Comprehensive molecular characterization of human colon and rectal cancer. Nature 2012; 487(7407):330–337.

30. Grabsch H, Dattani M, Barker L et al. Expression of DNA double-strand break repair proteins ATM and BRCA1 predicts survival in colorectal cancer. Clin. Cancer Res. 2006; 12(5):1494–1500.

36. Liu Y, Sethi NS, Hinoue T et al. Comparative Molecular Analysis of Gastrointestinal Adenocarcinomas. Cancer Cell 2018; 33(4):721–735.e8.

42. Schulpen M, van den Brandt PA. Mediterranean diet adherence and risk of colorectal cancer: the prospective Netherlands Cohort Study. Eur. J. Epidemiol. 2020; 35(1):25–35.

43. Brink M, Weijenberg MP, De Goeij AFPM et al. Fat and K-ras mutations in sporadic colorectal cancer in The Netherlands Cohort Study. Carcinogenesis 2004; 25(9):1619–1628.

44.  Brink M, de Goeij AFPM, Weijenberg MP et al. K-ras oncogene mutations in sporadic colorectal cancer in The Netherlands Cohort Study. Carcinogenesis 2003; 24(4):703–710.
